# Synthesis and Fundamental Evaluation of Radioiodinated Rociletinib (CO-1686) as a Probe to Lung Cancer with L858R/T790M Mutations of Epidermal Growth Factor Receptor (EGFR)

**DOI:** 10.3390/molecules25122914

**Published:** 2020-06-24

**Authors:** Muammar Fawwaz, Kenji Mishiro, Ryuichi Nishii, Izumi Sawazaki, Kazuhiro Shiba, Seigo Kinuya, Kazuma Ogawa

**Affiliations:** 1 Graduate School of Medical Sciences, Kanazawa University, Kakuma-machi, Kanazawa, Ishikawa 920-1192, Japan; muammar.fawwaz@stu.kanazawa-u.ac.jp (M.F.); izumisw@stu.kanazawa-u.ac.jp (I.S.); kinuya@med.kanazawa-u.ac.jp (S.K.); 2 Faculty of Pharmacy, Universitas Muslim Indonesia, Urip Sumoharjo KM. 10, Makassar 90-231, Indonesia; 3 Institute for Frontier Science Initiative, Kanazawa University, Kakuma-machi, Kanazawa, Ishikawa 920-1192, Japan; mishiro@p.kanazawa-u.ac.jp; 4 National Institute of Radiological Sciences (NIRST), QST, Inage-ku, Chiba 263-8555, Japan; nishii.ryuichi@qst.go.jp; 5 Advanced Science Research Center, Kanazawa University, Takara-machi, Kanazawa, Ishikawa 920-8640, Japan; shiba@med.kanazawa-u.ac.jp

**Keywords:** imaging, EGFR, prediction of therapeutic effects, mutation status, radiopharmaceutical, tyrosine kinase inhibitor

## Abstract

Rociletinib (CO-1686), a 2,4-diaminopyrimidine derivative, is a highly potent tyrosine kinase inhibitor (TKI) that acts on epidermal growth factor receptor (EGFR) with L858R/T790M mutations. We supposed radioiodinated CO-1686 would function as a useful tool for monitoring EGFR L858R/T790M mutations. To aid in patient selection before therapy with EGFR-TKIs, this study aimed to develop a ^125^I-labeled derivative of CO-1686, *N*-{3-[(2-{[4-(4-acetylpiperazin-1-yl)-2-methoxyphenyl]amino}-5-(trifluoromethyl)pyrimidine-4-yl] amino}-5-([^125^I]iodophenyl)acrylamide ([^125^I]ICO1686) and evaluate its selectivity toward EGFR L858R/T790M. Radiosynthesis was performed by iododestannylation of the corresponding tributylstannyl precursor with [^125^I]NaI and *N*-chlorosuccinimide. The selectivity of the tracer for detecting EGFR L858R/T790M was evaluated using three relevant non-small cell lung cancer (NSCLC) cell lines—H1975, H3255 and H441 overexpressing the dual mutation EGFR L858R/T790M, active mutant EGFR L858R and wild-type EGFR, respectively. The nonradioactive ICO1686 and the precursor compound were successfully synthesized. A novel radiolabeled probe, [^125^I]ICO1686, was prepared with high radiochemical yield (77%) and purity (>99%). ICO1686 exhibited high cytotoxicity toward H1975 (IC_50_ 0.20 ± 0.05 μM) and H3255 (IC_50_ 0.50 ± 0.21 μM), which is comparable to that of CO-1686. In contrast, the cytotoxicity of ICO1686 toward H441 was 10-fold lower than that toward H1975. In the cell uptake study, the radioactivity uptake of [^125^I]ICO1686 in H1975 was 101.52% dose/mg, whereas the uptakes in H3255 and H441 were 33.52 and 8.95% dose/mg, respectively. The uptake of [^125^I]ICO1686 in H1975 was greatly reduced to 45.61% dose/mg protein by treatment with excess CO-1686. In vivo biodistribution study of the radiotracer found that its accumulation in H1975 tumor (1.77 ± 0.43% ID/g) was comparable to that in H3255 tumor (1.63 ± 0.23% ID/g) and the accumulation in H1975 tumor was not reduced by pretreatment with an excess dose of CO-1686. Although this radiotracer exhibited highly specific in vitro uptake in target cancer cells, structural modification is required to improve in vivo biodistribution.

## 1. Introduction

Despite the continuous development of cancer treatments, lung cancer remains a deadly disease worldwide [1]. Non-small cell lung cancer (NSCLC) is the most prevalent type, accounting for over 80% of all lung cancers [2,3]. Activated mutations in exons 18–21, in the kinase domain of epidermal growth factor receptor (EGFR), were associated with NSCLC; among them, frequently occurring mutations are the single L858R mutation in exon 21 and the EGFR exon 19 deletion [4,5,6].

The treatment of patients with NSCLC with EGFR-tyrosine kinase inhibitors (TKIs) has shown impressive therapeutic outcomes [3,7]. The first-generation EGFR-TKIs, gefitinib and erlotinib, significantly prolonged the overall survival of patients with NSCLC with the EGFR exon 19 deletion or EGFR L858R mutation [8,9]. However, most patients acquire a further mutation in EGFR after long-term drug administration of first-generation EGFR-TKIs. Drug resistance eventually develops. T790M in exon 20 was identified as the most frequent cause of resistance [7,10]. Some third-generation EGFR-TKIs were designed to overcome drug resistance, among which osimertinib (Tagrisso™) was approved by the Food and Drug Administration (FDA) for the treatment of patients with NSCLC with EGFR L858R/T790M mutations [8,9,11].

Currently, biopsy is a common strategy for detecting EGFR mutations in NSCLC. Biopsy samples provide information concerning cancer genetics through gene sequencing, immunohistochemistry and fluorescence in situ hybridization (FISH) analysis [12,13]. However, the analysis of biopsy specimens does not necessarily reflect complete tumor mutation status because tumor tissues are heterogenous and the tiny fraction of them in a biopsy may not be representative of the whole tumor profile [14,15]. Additionally, repeated biopsies are invasive and a significant burden on patients [12,13]. In contrast, nuclear medicine imaging, such as positron emission tomography (PET) and single-photon emission computed tomography (SPECT), is a revolutionary invention in the field of clinical diagnostics because of its non-invasive character and great ability to determine the expression levels of target molecules [13,16].

To effectively use third-generation EGFR-TKIs on patients with NSCLC with EGFR L858R/T790M mutations [8,17], we hypothesized that PET or SPECT imaging would be a suitable and non-invasive tool for selecting patients who are sensitive to third-generation EGFR-TKIs. Radiotracers based on the third-generation EGFR-TKIs, such as [^11^C]osimertinib and [^11^C]rociletinib ([^11^C]CO-1686), were developed to display the brain penetration of TKIs in a preclinical setting [11,18]; however, these studies were not designed for the selection of patients sensitive to EGFR L858R/T790M TKIs. Until now, although some molecular probes for the L858R mutation were developed no reports exist [19,20], to our knowledge, on molecular probes with sufficient sensitivity for L858R/T790M double mutations and the development of such probes is anticipated.

CO-1686, a third-generation EGFR-TKI, is a mutant-selective, irreversible inhibitor of EGFR mutations, including exon 19 deletions, L858R and T790M but not exon 20 insertions [21]. CO-1686 exhibited striking potency against EGFR with L858R/T790M mutations in in vitro studies and clinical trials [22,23]. Meanwhile, preclinical data have confirmed that CO-1686 shows low activity against wild-type EGFR [21,22,23].

**Scheme 1:**
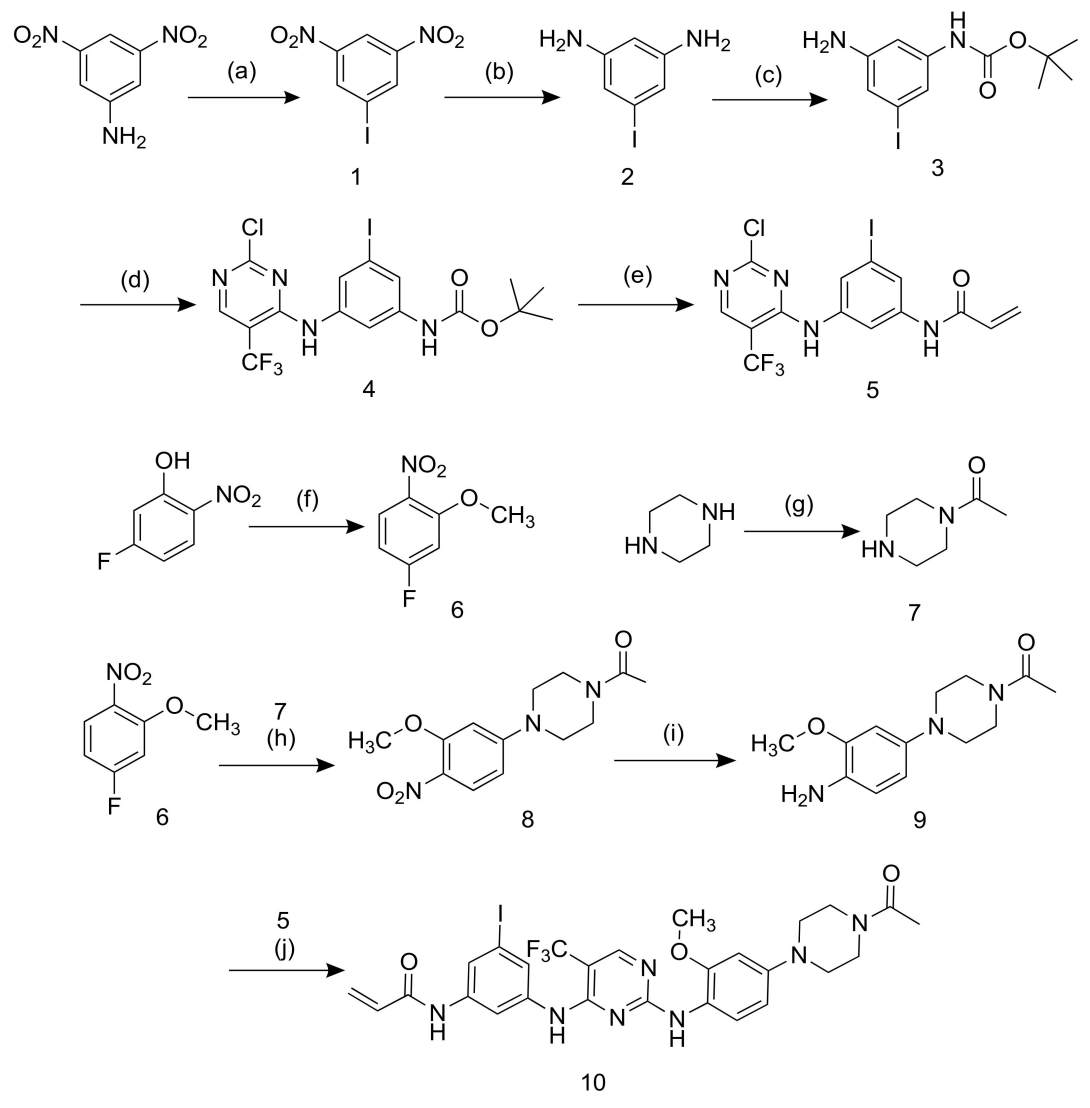
Synthetic scheme of non-radioactive iodinated compound. Reagent: (a) H_2_SO_4_, NaNO_2_, KI, 0 °C, rt, 3 h; (b) Ethanol, SnCl_2_·2H_2_O, 45 °C, 1 h; (c) Acetonitrile, Boc_2_O, rt, 2 h; (d) *n*-butanol, 2,4-dichloro-5(trifluoromethyl) pyrimidine, DIPEA, 0 °C, 1 h, rt, 4 h; (e) HCl/ethyl acetate, rt, 1 h, dichloromethane, acryloyl chloride, DIPEA, −30 °C, rt, 1 h; (f) Acetone, K_2_CO_3_, CH_3_I, 0 °C, 5 h; (g) Dichloromethane, acetic anhydride, 0 °C, 5 h; (h) DMA, DIPEA, 90 °C, overnight; (i) Pd/C 10%, H_2_, rt, 5 h; (j) Dioxane, TFA, 60 °C, 3 h.

**Scheme 2:**
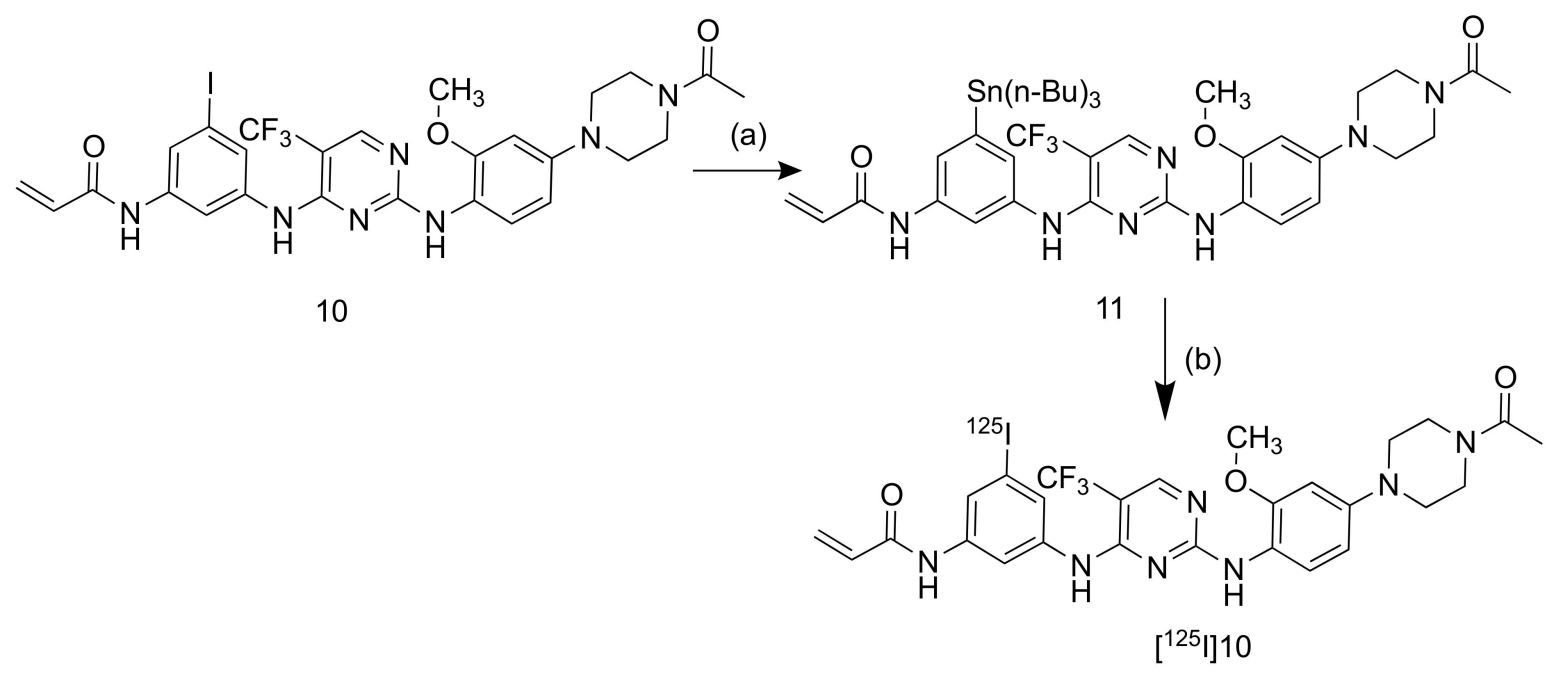
Synthesis of tin precursor **11** and radioiodinated compound [^125^I]**10**. (a) Dry dioxane, hexabutyldistannane, PdCl_2_(PPh_3_)_2_, 60 °C, 18 h; (b) [^125^I]NaI, acetic acid, NCS, 37 °C, 15 min.

In this study, we designed and synthesized a non-radioactive iodinated, *N*-(3-{[2-({4-[4-acetylpiperazin-1-yl]-2-methoxyphenyl}amino)-5-(trifluoromethyl)pyrimidin-4-yl]amino}-5-iodophenyl)acrylamide (ICO1686, Scheme 1, **10**) and a radioactive iodinated **10** ([^125^I]ICO1686, Scheme 2, [^125^I]**10**), and evaluated it using three types of human NSCLC cell lines—H1975 (dual mutations EGFR L858R/T790M), H3255 (EGFR L858R active mutant) and H441 (wild-type EGFR)—for use in selecting patients with NSCLC and predicting treatment strength of third-generation EGFR-TKIs. In this study, although we aimed at developing probes for PET (^124^I) or SPECT (^123^I), ^125^I was used as an alternative radionuclide for initial fundamental studies because of its longer half-life.

## 2. Results and Discussion

### 2.1. Synthesis of Non-Radioactive Compound

The non-radioactive iodinated compound **10**, a derivative of CO-1686, was synthesized according to the reported synthesis procedure of CO-1686 starting from 5-iodo-*m*-phenylenediamine instead of *m*-phenylenediamine (Scheme 1). 5-Iodo-*m*-phenylenediamine was synthesized in two steps from commercially available 1,3-dinitroaniline. Iodine was introduced to the diaminophenyl group of CO-1686, because this substituent would not overly influence the affinity of CO-1686 for EGFR L858R/T790M due to the complex crystal structure of CO-1686 and T790M EGFR [7]. Their complex crystal structure suggests that the anilinopyrimidine core, methoxy and trifluoromethyl groups are the most important substituents and play a key role in the binding of CO-1686 to EGFR L858R/T790M [7].

### 2.2. Cell Viability Assays

As displayed in Table 1, the cytotoxicity of **10** toward H1975 and H3255 were similar to those of CO-1686, whereas the cytotoxicity of **10** toward H441 was significantly lower than that of non-iodinated CO-1686. These results demonstrate that the iodine substituent in **10** did not significantly affect activity toward H1975 with L858R/T790M EGFR and H3255 with L858R EGFR mutations. In this study, the introduction of iodine reduced the cytotoxicity of **10** toward H441; however, this was unimportant because CO-1686 is indeed less potent against wild-type than L858R/T790M mutations [20,24]. Conversely, gefitinib exhibited high cytotoxicity toward only H3255 cells. All of these results are consistent with previous reports and clinical data [19,20,21,22,23]. For example, the IC_50_ value of CO-1686 to H1975 (0.14 µM) in this study is consistent with the value (0.137 μM) in the previous study [25].

In our study, CO-1686 showed moderately inhibitory activity toward a human lung cancer cell line with a wild-type EGFR (H441). Walter et al. reported that CO-1686, which did not inhibit wild-type EGFR kinase activity, exhibited moderate cytotoxicity toward a cancer cell line with wild-type EGFR [24]. Although the mechanism of cytotoxicity is unclear, the cytotoxicity of CO-1686 toward H441 observed in our study may be associated with CO-1686 drug activity not related to EGFR.

### 2.3. EGFR-Tyrosine Kinase (TK) Inhibition Assay

Inhibition activities of CO-1686 and **10** to EGFR-TK were evaluated. IC_50_ values to EGFR-TK with L858R/T790M mutation and wild-type EGFR-TK are shown in Table 2. CO-1686 inhibited EGFR L858R/T790M enzyme activity at low concentration (IC_50_ = 0.04 μM). The IC_50_ value is consistent with that in a previous report (IC_50_ = 0.02 μM) [25]. Although the inhibition activity of **10** to L858R/T790M (IC_50_ = 0.31 μM) was weaker than that of CO-1686, **10** exhibited high selectivity index (SI > 32) of EGFR with L858R/T790M double mutations to wild-type EGFR. It was comparable to that of CO-1686 (SI = 42). These results indicate that not only CO-1686 but also **10** has high specificity toward EGFR with L858R/T790M double mutations.

### 2.4. Radiolabeling of [^125^I]ICO1686 ([^125^I]10)

A stannylated radiolabeling precursor **11** was prepared by a palladium-catalyzed stannylation reaction of **10** in 10% yield after purification using high-performance liquid chromatography (HPLC). As provided in Scheme 2**,** [^125^I]**10** was synthesized by an iododestannylation reaction with the corresponding tributyltin precursor **11**. This radiotracer was synthesized using *N*-chlorosuccinimide (NCS) as an oxidizing agent in an acidic solution at 37 °C in high radiochemical yield (77%). Radiochemical purity was over 99% after purification using RP-HPLC. The identity of [^125^I]**10** was confirmed by comparing the retention times of the radioiodinated compound and nonradioactive **10** in the HPLC analyses. These peaks were shown at the same retention time in the chromatograms (Figure S12).

### 2.5. Determination of Partition Coefficient

The *n*-octanol/phosphate buffer partition coefficients (log *P*) values are one explanation for how [^125^I]**10** behaves in the body. The log *P* value indicating lipophilicity is significant in terms of membrane permeability of radiotracer into the intracellular domain, such as at an EGFR-TK site. The shake-flask method by phase separation resulting in the log *P* value for [^125^I]**10** was 1.84 ± 0.01. This result indicates that [^125^I]**10** exhibits appropriate lipophilicity to passively penetrate the cell membrane [26].

### 2.6. In Vitro Stability Experiments

The stability profile of [^125^I]**10** in phosphate-buffered saline (PBS) and murine plasma is shown in the Supporting Information (Figure S13). Until 1 h, [^125^I]**10** slightly decomposed in either the PBS or plasma and its purity decreased gradually after 24 h incubation.

### 2.7. Cellular Uptake Studies

**Figure 1:**
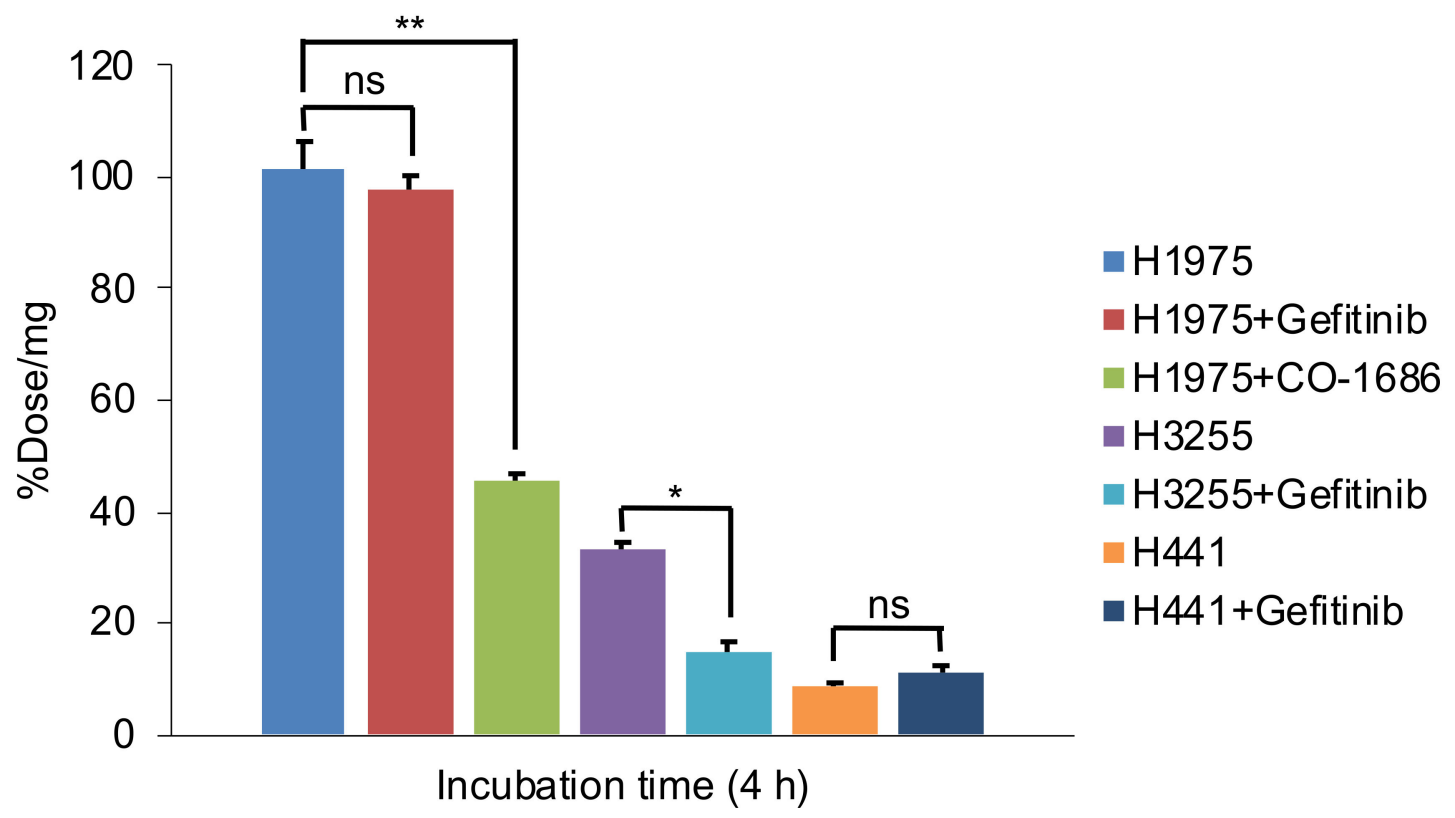
The cell uptake of radiolabeled compound [^125^I]**10** to the H1975, H3255 and H441 cell lines. Significance was determined using Dunnett’s multiple-comparison test or unpaired Student’s *t*-test (* *p* < 0.001; ** *p* < 0.0001) ns: not significant.

To investigate the differences in [^125^I]**10** uptake based on EGFR mutational and/or expression status, we performed in vitro cell uptake experiments using three types of NSCLC cell lines—H1975 (L858R/T790M active mutant EGFR), H3255 (L858R active mutant EGFR) and H441 (wild-type EGFR). As shown in Figure 1, H1975 cells exhibited a high accumulation of [^125^I]**10,** at 101.52% dose/mg protein at 4 h incubation. The accumulation was significantly higher than that in H3255 and H441 cells at 33.52 and 8.95% dose/mg protein, respectively. The presence of the T790M activating mutation in the EGFR kinase domain accounts for the greater accumulation of [^125^I]**10** in H1975 cells [21,22].

In fact, drug resistance is caused by EGFR L858R/T790M mutations that result in threonine-to-methionine substitution at residue 790, namely the gatekeeper residue (T790M). These mutations do not only enhance the affinity of the kinase for adenosine triphosphate (ATP) but also decrease the affinity with TKIs, such as gefitinib and erlotinib [7]. Therefore, CO-1686, with its unique structure, was developed to encounter this resistance. The anilinopyrimidine core of CO-1686 forms bidentate hydrogen bonds with the main chain NH and CO of Met793 in the EGFR kinase domain. The methoxyl group contributes to the selectivity of CO-1686 for EGFR. In addition, the trifluoromethyl substituent of CO-1686 is conducive to forming hydrophobic interactions with Met790 as the mutant gatekeeper residue [7]. This hydrophobicity is only accorded by Met790 which is not accorded by wild-type gatekeeper residue (Thr790) [7]. This explains why [^125^I]**10** prefers to accumulate in H1975 expressing EGFR L858R/T790M mutations.

In the blocking studies, the uptakes of [^125^I]**10** in H1975 were significantly reduced to 45.61% dose/mg protein by pretreatment with an excess of CO-1686, indicating that [^125^I]**10** bound competitively, versus CO-1686, to EGFR-TK with L858R/T790M double mutations. However, the degree of the blocking effects was lower than we expected. It means that the nonspecific binding of [^125^I]**10** on or inside H1975 cells may be not low. Although H1975 cells harbor the L858R EGFR mutation, gefitinib did not reduce the accumulation of [^125^I]**10** in in vitro blocking experiments. This was due to the T790M mutation, which interferes with the binding of gefitinib to the tyrosine kinase domain of EGFR, thereby conferring resistance to gefitinib [20]. In contrast, the radiotracer accumulation of [^125^I]**10** in H3255 was dramatically reduced (over 50%) by pretreatment with a dose of gefitinib, which is attributable to the high affinity for gefitinib of H3255 cells carrying the L858R mutation [19,27,28].

### 2.8. Biodistribution Studies

Biodistribution experiments were performed in order to determine the in vivo accumulation of [^125^I]**10** in tissues. The biodistribution data in normal mice are summarized in Table 3. The high radioactivity rapidly accumulated in liver after the injection of [^125^I]**10**. This was considered to have been caused by the relatively hydrophobic structure of [^125^I]**10** (Log *P* 1.84 ± 0.01). Liver activity decreased gradually and [^125^I]**10** was transferred to the small intestine via bile excretion [29] and then to large intestine as time passed; as a result, the large intestine exhibited high uptake of the radiotracer (53.44 ± 6.73% ID/g) at 4 h post injection. Finally, almost 70% of the radiotracer was excreted in feces 24 h after injection. Namely, [^125^I]**10** was mainly eliminated through hepatobiliary clearance. Meanwhile, Ballard et al. reported a PET image of [^11^C]CO-1686 in a monkey [11]. In the PET image using average data from 0 to 123 min, [^11^C]CO-1686 showed high accumulation in the intestine and moderate accumulation in the liver. Moreover, [^11^C]CO-1686 hardly accumulated in the brain [11]. These results seem to be similar to the biodistribution pattern of [^125^I]**10**.

Table 4 summarizes the biodistribution data in tumor-bearing mice inoculated with H1975 and H3255 cells. The accumulation of [^125^I]**10** in H1975 tumors (1.77 ± 0.43% ID/g) was comparable to that in H3255 tumors (1.63 ± 0.23% ID/g). Our data indicate that the in vivo uptake in mouse xenografts did not correlate with the in vitro uptake of [^125^I]**10** toward EGFR double mutations. Moreover, accumulation in H1975 tumors was not inhibited by pretreatment with an excess dose of CO-1686. In some cases, the blocking dose did not significantly inhibit radiotracer accumulation in tumors. Zhang et al. reported that gefitinib did not sufficiently block the uptake of [^11^C]gefitinib. In this case, it may have been due to high nonspecific binding in tumor regions [30].

As above-mentioned, the purpose of our study is the development of probes for PET or SPECT imaging using ^124^I or ^123^I. However, it must be difficult to visualize the tumor for [^123/124^I]**10,** even if the imaging would be performed using tumor-bearing mice because tumor/blood ratio was approximately 1. Moreover, tumor/lung ratio is also important for imaging of NSCLC patients in clinical. Therefore, an increase of tumor uptake and/or decrease of non-target tissue uptake to be background, such as lung and blood, are necessary.

Lack of specific tumor uptake of [^125^I]**10** was likely not caused by metabolic instability in blood, as we found that [^125^I]**10** hardly decomposed in the plasma until 1 h in the in vitro stability experiment. The high level of nonspecific uptake in normal tissues and the high hepatobiliary excretion are the possible reasons why tumor uptake was suboptimal [30]. The high lipophilicity was related to the hepatobiliary excretion of [^125^I]**10**, resulting in very high elimination of radiotracer, which might have influenced the intratumoral uptake of [^125^I]**10 [31]**. Improving water solubility may be necessary to improve the accumulation of [^125^I]**10** in tumors. Structural modification by a short-chain polyethylene glycol in the linker phenyl group may be an alternative for reducing nonspecific accumulation without a significant reduction of the affinity for EGFR L858R/T790M [7,32,33].

## 3. Materials and Methods

### 3.1. General Chemistry

All chemical reagents and solvents were purchased commercially from Tokyo Chemical Industry, Co., Ltd. (Tokyo, Japan), Merck (Darmstadt, Germany), Nacalai Tesque, Inc. (Kyoto, Japan), Fujifilm Wako Pure Chemical Corporation (Osaka, Japan) and Kanto Chemical, Co., Inc. (Tokyo, Japan). [^125^I]Sodium iodide (644 GBq/mg) was purchased from Perkin Elmer (Waltham, MA, USA). The radioactivity was measured by an Auto Gamma System ARC-7010B (Hitachi, Ltd., Tokyo, Japan). Reactions were performed at respective temperature and monitored by thin-layer chromatography (TLC) on silica plates 60 F_254_ (Merck, Darmstadt, Germany).

Dulbecco’s Modified Eagle Medium (DMEM)/Ham’s F-12 and RPMI-1640 medium, penicillin-streptomycin, phosphate-buffered saline (PBS) and trypsin–EDTA (0.25%) were obtained from Nacalai Tesque, Inc. (Kyoto, Japan). Fetal bovine serum (FBS) was obtained from Biowest (Nuaillé, France).

Nuclear magnetic resonance (NMR) spectroscopy was obtained on JEOL JNM-ECS 400 and JEOL JNM-ECA 600 (JEOL Ltd., Tokyo, Japan). Direct analysis in real time mass spectra (DART-MS) was obtained on JEOL JMS-T100TD (JEOL Ltd., Tokyo, Japan). Fast atom bombardment mass spectra (FAB-MS) was obtained on JEOL JMS-T700 (JEOL Ltd., Tokyo, Japan). Analytical high-performance liquid chromatography (HPLC) system was obtained on the Shimadzu SPD-20A system (Shimadzu Corp., Kyoto, Japan). The optical density in WST-8 assay and luminescence density in kinase assay were obtained on Infinite^®^ F200 Pro microplate reader (TECAN, Männedorf, Switzerland).

### 3.2. Synthesis Scheme

Intermediate compounds were synthesized according to the outlined studies, with slight modifications [34,35,36,37,38,39]. Detailed procedures and analysis were shown in Scheme 1 and Supporting Information (Figures S1–S11).

#### 3.2.1. Synthesis of 1-Iodo-3,5-dinitrobenzene (**1**)

To a stirred mixture of 3,5-dinitroaniline (C_6_H_5_N_3_O_4_) (0.70 g, 3.82 mmol, 1.0 eq.) and sulfuric acid (H_2_SO_4_) (0.60 mL, 11.46 mmol, 3.0 eq.), sodium nitrite (NaNO_2_) (0.40 g, 5.73 mmol, 1.5 eq.) was added at 0 °C. After the formation of the diazonium salt was confirmed by TLC, potassium iodide (KI) (1.60 g, 9.55 mmol, 2.5 eq.) was added to the mixture and the mixture was stirred at room temperature for 3 h. After the reaction was completed, pH was adjusted to neutral using saturated aqueous sodium bicarbonate (NaHCO_3_). Then the reaction mixture was extracted with ethyl acetate. The organic layer was separated, dried over sodium sulfate (Na_2_SO_4_), filtered and concentrated under reduced pressure to afford **1** (1.1 g, 97%) as a brown crystal. ^1^H–NMR (400 MHz, CDCl_3_): δ 8.88 (2H, d, *J* = 1.6 Hz), 9.02 (1H, t, *J* = 2.0 Hz). ^13^C–NMR (100 MHz, CDCl_3_): δ 93.5, 118.5, 137.8, 148.5. HRMS (FAB+) calculated for C_6_H_3_IN_2_O_4_ [M + H]^+^: *m/z* = 294.9216, found 294.9225.

#### 3.2.2. Synthesis of 5-Iodobenzene-1,3-diamine (**2**)

To a stirred mixture of **1** (1.10 g, 3.74 mmol, 1.0 eq.) and ethanol (20 mL), tin (II) chloride dihydrate (SnCl_2_·2H_2_O) (4.22 g, 18.70 mmol, 5.0 eq.) was added at 45 °C and the mixture was stirred for 1 h under nitrogen atmosphere. After the reaction was completed, the pH was adjusted to neutral using saturated aqueous sodium bicarbonate (NaHCO_3_). The reaction mixture was extracted with ethyl acetate. The organic layer was separated, dried over sodium sulfate (Na_2_SO_4_), filtered and concentrated under reduced pressure to afford **2** (0.62 g, 71%) as a brown solid. ^1^H–NMR (400 MHz, CDCl_3_): δ 3.57 (4H, s), 5.95 (1H, t, *J =* 2.0 Hz), 6.48 (2H, d, *J* = 2.0 Hz). ^13^C–NMR (100 MHz, CDCl_3_): δ 95.5, 101.0, 114.8, 148.5. HRMS (FAB+) calculated for C_6_H_7_IN_2_ [M + H]^+^: *m/z* = 233.9654, found 233.9645.

#### 3.2.3. Synthesis of *tert*-Butyl (3-amino-5-iodophenyl)carbamate (**3**)

To a stirred mixture of **2** (0.62 g, 2.65 mmol, 1.0 eq.) and acetonitrile (5.0 mL), di-*tert*-butyl dicarbonate (Boc_2_O) (0.60 g, 2.65 mmol, 1.0 eq.) was added at room temperature and the mixture was stirred for 2 h under nitrogen atmosphere. After the reaction was completed, the product was purified by column chromatography on silica gel (hexane/ethyl acetate = 7/3) and concentrated under reduced pressure to afford **3** (0.28 g, 32%) as an orange crystal. ^1^H–NMR (400 MHz, CDCl_3_): δ 1.50 (9H, s), 3.68 (2H, s), 6.34 (1H, s), 6.71 (1H, t, *J* = 1.6 Hz), 6.86 (1H, s), 6.96 (1H, t, *J* = 1.6 Hz). ^13^C–NMR (100 MHz, CDCl_3_): δ 28.5 (3C), 80.9, 94.8, 104.0, 117.2, 118.7, 140.3, 148.3, 152.5. HRMS (FAB+) calculated for C_11_H_15_IN_2_O_2_ [M + H]^+^: *m/z* = 334.0178, found 334.0168.

#### 3.2.4. Synthesis of *tert*-Butyl (3-{[2-chloro-5-(trifluoromethyl)pyrimidin-4-yl]amino}-5-iodophenyl) carbamate (**4**)

To a stirred mixture of **3** (0.28 g, 0.85 mmol, 1.0 eq.) and *n*-butanol (2.6 mL), 2,4-dichloro-5(trifluoromethyl)pyrimidine (115 μL, 0.85 mmol, 1.0 eq.), *N*,*N*-diisopropylethylamine (DIPEA) (296 μL, 1.70 mmol, 2.0 eq.) were added at 0 °C and stirred for 1 h. The stirring was continued at room temperature for 4 h. After the reaction was completed, the crude product was purified using column chromatography on silica gel (dichloromethane) and concentrated under reduced pressure to afford **4** (0.29 g, 66%) as a colorless solid. ^1^H–NMR (400 MHz, CDCl_3_): δ 1.53 (9H, s), 6.68 (1H, s), 7.00 (1H, s), 7.58 (1H, t, *J* = 1.6 Hz), 7.61 (1H, t, *J* = 1.6 Hz), 7.72 (1H, t, *J* = 1.6 Hz), 8.46 (1H, s). ^13^C–NMR (100 MHz, CDCl_3_): δ 28.2 (3C), 81.4, 93.8, 106.7 (q, *J_CF_* = 31.4 Hz), 111.6, 123.0 (q, *J_CF_* = 270 Hz), 124.2, 125.4, 137.6, 140.1, 152.2, 156.1 (q, *J_CF_* = 4.7 Hz), 157.0, 163.5. HRMS (FAB+) calculated for C_16_H_15_ClF_3_IN_4_O_2_ [M + H]^+^: *m/z* = 513.9880, found 513.9875.

#### 3.2.5. Synthesis of *N*-(3-{[2-chloro-5-(trifluoromethyl)pyrimidin-4-yl]amino}-5-iodophenyl) acrylamide (**5**)

The mixture of compound **4** (1.0 g, 1.94 mmol, 1.0 eq.) and 4.0 N HCl/ethyl acetate (1.0 mL) was stirred at room temperature for 1 h. The reaction was monitored by TLC (hexane/ethyl acetate = 7/3) after the reaction was completed, the solvent was removed by nitrogen gassing and the residue was dried under reduced pressure to afford a colorless solid (0.81 g). The crude material was used to the next step without further purification. To a stirred mixture of the crude material (0.81 g), *N*, *N*-diisopropylethylamine (DIPEA) (1.0 mL, 5.85 mmol, 3.0 eq.) and dichloromethane (20 mL), acryloyl chloride (237 μL, 2.93 mmol, 1.5 eq.) were added at ‒30 °C and stirred at room temperature for 1 h. The crude product was dissolved in ethyl acetate and washed with 1.0 M aqueous hydrochloric acid (HCl), saturated aqueous sodium bicarbonate (NaHCO_3_) and brine successively. The organic layer was separated, dried over sodium sulfate (Na_2_SO_4_), filtered and concentrated under reduced pressure. The crude product was purified using column chromatography on silica gel (hexane/ethyl acetate = 7/3) and concentrated under reduced pressure to afford **5** (380 mg, 43%) as a colorless solid. ^1^H NMR (400 MHz, (CD_3_)_2_SO): δ 5.79 (1H, dd, *J* = 10.0, 2.0 Hz), 6.27 (1H, dd, *J* = 16.8, 2.0 Hz), 6.40 (1H, dd, *J* = 16.4, 10.0 Hz), 7.56 (1H, d, *J* = 2.0 Hz), 7.77 (1H, d, *J* = 2.0 Hz), 7.99 (1H, d, *J* = 2.0 Hz), 8.62 (1H, s), 9.57 (1H, s), 10.31 (1H, s). ^13^C NMR (150 MHz, (CD_3_)_2_SO): δ 93.6, 106.1 (q, *J_CF_* = 30.2 Hz), 115.8, 123.0 (q, *J_CF_* = 270 Hz), 125.0, 127.7, 128.9, 131.4, 138.4, 140.4, 156.9 (q, *J_CF_* = 4.4 Hz), 157.5, 162.3, 163.4. HRMS (FAB+) calculated for C_14_H_9_ClF_3_IN_4_O [M + H]^+^: *m/z* = 468.9461, found 468.9559.

#### 3.2.6. Synthesis of 4-Fluoro-2-methoxy-1-nitrobenzene (**6**)

To a stirred mixture of 5-fluoro-2-nitrophenol (C_6_H_4_FNO_3_) (1.0 g, 6.43 mmol, 1.0 eq.) and acetone (15 mL), potassium carbonate (K_2_CO_3_) (1.77 g, 12.80 mmol, 2.0 eq.) and iodomethane (CH_3_I) (0.80 mL, 12.80 mmol, 2.0 eq.) were added. The mixture was stirred at 0 °C for 5 h. After the reaction was completed, ethyl acetate and water (1:1) were added to the mixture. The organic layer was separated, dried over sodium sulfate (Na_2_SO_4_), filtered and concentrated under reduced pressure to afford **6** (1.1 g, 100%) as an orange crystal. ^1^H–NMR (400 MHz, CDCl_3_): δ 3.97 (3H, s), 6.74 (1H, dt, *J =* 8.4, 2.0 Hz), 6.80 (1H, dd, *J =* 10.4, 2.4 Hz), 7.96 (1H, dd, *J* = 8.4, 6.4 Hz). ^13^C–NMR (100 MHz, (CD_3_)_2_SO): δ 56.7, 101.5, 107.0, 128.0, 155.3, 164.3, 167.0. HRMS (DART+) calculated for C_7_H_6_FNO_3_ [M + H]^+^: *m/z* = 172.0331, found 172.0345.

#### 3.2.7. Synthesis of 1-(Piperazine-1-yl)ethan-1-one (**7**)

To a stirred mixture of piperazine (C_4_H_10_N_2_) (1.70 g, 19.70 mmol, 2.0 eq.) and dichloromethane (50 mL), acetic anhydride (C_4_H_6_O_3_) (0.94 mL, 9.90 mmol, 1.0 eq.) was added at 0 °C and the mixture was stirred for 5 h. After the reaction was completed, the reaction mixture was quenched with water (15 mL) and extracted with dichloromethane, the organic layer then dried over sodium sulfate (Na_2_SO_4_), filtered and concentrated under reduced pressure to afford **7** (0.26 g, 20%) as a colorless solid. ^1^H–NMR (400 MHz, CDCl_3_): δ 2.13 (3H, s), 3.40–3.70 (8H, m). ^13^C–NMR (100 MHz, CDCl_3_): δ 21.3, 41.2 (2C), 45.7 (2C), 169.1. HRMS (FAB+) calculated for C_6_H_12_N_2_O [M + H]^+^: *m/z* = 129.0949, found 129.1031.

#### 3.2.8. Synthesis of 1-(4-{3-Methoxy-4-nitrophenyl}piperazine-1-yl)ethan-1-one (**8**)

To a stirred mixture of **7** (1.90 g, 11.0 mmol, 1.0 eq.) and dimethylacetamide (DMA) (6.0 mL), **6** (1.40 g, 11.0 mmol, 1.0 eq.) and *N*,*N*-diisopropylethylamine (DIPEA) (2.0 mL) were added at 90 °C. The mixture was stirred overnight under nitrogen atmosphere with a reflux condenser. After the reaction was completed, the reaction mixture was quenched with water and extracted with ethyl acetate. The organic layer was dried over sodium sulfate (Na_2_SO_4_), filtered and concentrated under reduced pressure. The crude product was purified using column chromatography on silica gel (chloroform/methanol = 150/1) to afford **8** (2.61 g, 83%) as a pale brown solid. ^1^H–NMR (400 MHz, CDCl_3_): δ 2.16 (3H, s), 3.40–3.50 (4H, m), 3.67 (2H, t, *J* = 4.0 Hz), 3.81 (2H, t, *J* = 4.0 Hz), 3.97 (3H, s), 6.33 (1H, d, *J* = 1.6 Hz), 6.42 (1H, dd, *J* = 6.0, 1.6 Hz), 8.02 (1H, d, *J* = 6.4 Hz). ^13^C–NMR (100 MHz, CDCl_3_): δ, 21.2, 40.5, 45.2, 46.5, 46.6, 56.0, 97.0, 105.1, 128.4, 129.5, 155.0, 156.0, 169.0. HRMS (FAB+) calculated for C_13_H_17_N_3_O_4_ [M + H]^+^: *m/z* = 280.1219, found 280.1295.

#### 3.2.9. Synthesis of 1-(4-{4-Amino-3-methoxyphenyl}piperazine-1-yl)ethan-1-one (**9**)

To a stirred mixture of **8** (100 mg, 0.35 mmol, 1.0 eq.) and ethanol (3.50 mL) under a nitrogen atmosphere, palladium on carbon (Pd/C 10%) (20 mg) was added at room temperature. The mixture then stirred under a hydrogen atmosphere for 5 h. After the reaction was completed, the catalyst was removed by filtration through a pad of Celite^®^; the filtrate was concentrated under reduced pressure to afford **9** (98 mg, 100%) as a purple solid. This compound was so unstable that it was used in the following reaction without further purification right after characterization by ^1^H NMR and MS analyses. ^1^H–NMR (400 MHz, CDCl_3_): δ 2.13 (3H, s), 2.95–3.08 (4H, m), 3.62 (2H, t, *J* = 5.6 Hz), 3.77 (2H, t, *J* = 5.2 Hz), 3.85 (3H, s), 6.42 (1H, dd, *J =* 8.8, 2.8 Hz), 6.52 (1H, s), 6.66 (1H, d, *J =* 8.4 Hz). HRMS (FAB+) calculated for C_13_H_19_N_3_O_2_ [M + H]^+^: *m/z* = 249.1477, found 249.1464.

#### 3.2.10. Synthesis of *N*-(3-{[2-({4-[4-acetylpiperazin-1-yl]-2-methoxyphenyl}amino)-5- (trifluoromethyl)pyrimidin-4-yl]amino}-5-iodophenyl)acrylamide (10)

To a stirred mixture of compound **5** (25 mg, 0.05 mmol, 1.0 eq.) and 2.0 M TFA/dioxane, compound **9** was added (17 mg, 0.068 mmol, 1.36 eq.) and the mixture was stirred at 60 °C for 3 h. After the reaction was completed, the pH was adjusted to neutral using saturated aqueous sodium bicarbonate (NaHCO_3_). The reaction mixture was extracted with ethyl acetate. The organic layer was separated, dried over sodium sulfate (Na_2_SO_4_), filtered and concentrated under reduced pressure. The crude product was purified by HPLC with mobile phase system ethyl acetate/methanol = 97/3, using a Cosmosil^®^ 5SL-II (20 ID × 250 mm) column, flow rate 9.5 mL/min. The column temperature was maintained at 40 °C. The pure product was concentrated under reduced pressure to afford **10** (30 mg, 73%) as a colorless solid. ^1^H–NMR (400 MHz, (CD_3_)_2_SO): δ 2.05 (3H, s), 3.10 (2H, t, *J* = 4.8 Hz), 3.16 (2H, t, *J* = 4.4 Hz), 3.53–3.63 (4H, m), 3.82 (3H, s), 5.77 (1H, dd, *J* = 10.0, 2.0 Hz), 6.25 (1H, dd, *J* = 16.8, 2.0 Hz), 6.41 (1H, dd, *J* = 16.8, 10.0 Hz), 6.49 (1H, dd, *J* = 8.8, 2.0 Hz), 6.71 (1H, s), 7.74 (1H, s), 7.77 (2H, s), 7.82 (1H, s), 7.86 (1H, s), 8.35 (1H, s), 9.79 (1H, s), 10.14 (1H, s). ^13^C–NMR (100 MHz, (CD_3_)_2_SO): δ 21.0, 40.8, 45.4, 48.7, 49.0, 56.0, 94.1, 98.3 (q, *J_CF_* = 30.5 Hz), 100.8, 107.5, 110.9, 119.1, 122.2, 124.1, 124.5, 124.8 (q, *J_CF_* = 270 Hz), 127.4, 131.6, 140.1, 141.2, 149.0, 151.8, 155.2 (q, *J_CF_* = 4.8 Hz), 156.8, 160.8, 163.2, 168.3. HRMS (FAB+) calculated for C_27_H_27_F_3_IN_7_O_3_ [M + H]^+^: *m/z* = 681.1172, found 681.1186.

#### 3.2.11. Synthesis of *N*-(3-{[2-({4-[4-acetylpiperazin-1-yl]-2-methoxyphenyl}amino)-5- (trifluoromethyl)pyrimidin-4-yl]amino}-5-{tributylstannyl}phenyl)acrylamide (**11**)

To a stirred mixture of compound **10** (35 mg, 51.40 μmol, 1.0 eq.) and dry dioxane (5 mL), hexabutyldistannane (519 μL, 1.03 mmol, 20 eq.) and palladium(II)bis(triphenylphosphine) dichloride (PdCl_2_(PPh_3_)_2_) (15 mg, 21 μmol, 0.4 eq.) were added, the mixture was stirred at 60 °C for 18 h under nitrogen atmosphere. After the reaction was completed, the catalysis was removed by filtration through a pad of Celite^®^; the filtrate was concentrated under reduced pressure. The crude product was purified by HPLC with mobile phase system ethyl acetate/methanol = 97/3, using a Cosmosil^®^ 5SL-II (20 ID × 250 mm) column, flow rate 9.5 mL/min. The column temperature was maintained at 40 °C. The pure product was concentrated under reduced pressure to afford **11** (4 mg, 10%) as a white solid. ^1^H–NMR (400 MHz, (CDCl_3_): δ 0.88 (9H, t, *J* = 7.6 Hz), 1.06 (6H, t, *J* = 8.0 Hz), 1.33 (6H, sex, *J =* 7.6 Hz), 1.54 (6H, quin, *J* = 7.6 Hz), 2.14 (3H, s), 3.10–3.20 (4H, m), 3.62 (2H, t, *J* = 5.6 Hz), 3.78 (2H, t, *J* = 5.6 Hz), 3.91 (3H, s), 5.75 (1H, d, *J* = 11.2 Hz), 6.23 (1H, dd, *J* = 17.2, 10.8 Hz), 6.43 (1H, d, *J* = 1.6 Hz), 6.48 (1H, dd, *J* = 7.6, 2.0 Hz), 6.54 (1H, d, *J* = 2.4 Hz), 7.68 (1H, s) 7.06 (1H, s), 7.14 (1H, s), 7.20 (1H, s), 7.44 (1H, s), 7.97 (1H, s), 8.16 (1H, d, *J* = 8.4 Hz), 8.29 (1H, s). HRMS (FAB+) calculated for C_39_H_54_F_3_N_7_O_3_Sn [M + H]^+^: *m/z* = 845.3267 found 845.3262.

### 3.3. Cell Viability Assays (WST-8 Assay)

WST-assay was performed to evaluate the cytotoxicity of **10**, CO-1686 and gefitinib toward NSCLC cell lines. The NSCLC cell lines H1975, H441 and H3255 were kindly supplied by Dr. Juri G. Gelovani, formerly of the Department of Experimental Diagnostic Imaging, at the University of Texas, MD Anderson Cancer Center, Houston, TX, USA. H1975 (2.5 × 10^3^ cells/well), H441 (2.5 × 10^3^ cells/well) and H3255 (1 × 10^4^ cells/well) were seeded in 96-well tissue culture plates and cultured in medium supplemented with 10% fetal bovine serum (FBS) and penicillin (100 IU/mL) streptomycin (100 mg/mL) at 37 °C in a humidified atmosphere with 5% CO_2_. After incubation for 24 h, cells were treated with various concentrations (0.01–100 µM) of **10**, CO-1686 or gefitinib for 48 h and cell viability was assessed using the Cell Counting Kit-8 (Dojindo, Kumamoto, Japan) following the manufacturer’s protocol. All data were analyzed using GraphPad Prism 5.0 software (GraphPad Software, San Diego, CA., USA) and presented as mean ± standard deviation (SD).

### 3.4. Kinase Enzymatic Assays

The inhibition ability of **10** and CO-1686 on EGFR tyrosine kinase activity was performed using an ADP-Glo Lipid Kinase Assay kit (Promega Corporation, Madison, AL, USA). The experiments were carried out according to the instructions of the manufacturer. The test was performed in a 384-well plate and the luminescence density was determined on Infinite^®^ F200 Pro microplate reader. All data were analyzed using GraphPad Prism 5.0 software and presented as mean ± SD.

### 3.5. Radiosynthesis of [^125^I]ICO1686 ([^125^I]10)

Radiosynthesis was performed as in the previous study with a slight alteration [40]. [^125^I]**10** was synthesized by an iododestannylation reaction of the corresponding precursor **11** (Scheme 2). A solution of [^125^I]NaI in NaOH aqueous solution (2.5 MBq, 1 µL) was charged into a sealed vial containing **11** (1 mg/mL, 10 µL), acetic acid (5%, 30 µL) and NCS (20 mg/mL, 10 µL). The mixture was vortexed 5 min and shaken 15 min at 37 °C. The product was quenched by the addition of aqueous sodium hydrogen sulfite (5 mg/mL, 10 µL) and was analyzed by reversed-phase (RP)-HPLC with mobile phase system water (A) and methanol (B), B: 70–100%, 20 min using a Cosmosil^®^ 5C_18_ MSII (4.6 ID × 150 mm) column, a flow rate 1 mL/min. The column temperature was maintained at 40 °C. Radiochemical yields and purities were determined by an auto well gamma counter.

### 3.6. Determination of Partition Coefficient

The partition coefficient of [^125^I]**10** was measured, as previously reported with a slight modification [41]. Briefly, [^125^I]**10** was added to the mixture of each 3 mL of *n*-octanol and 0.1 M phosphate buffer (pH 7.4) in a test tube. The test tube was vortexed for 1 min, left at room temperature for 10 min and centrifuged at 3000× *g* and 4 °C for 5 min. The *n*-octanol layer (2 mL) was transferred into a new test tube followed by adding 1 mL of new *n*-octanol and 3 mL of phosphate buffer. The radioactivity of each 1 mL layer, *n*-octanol and phosphate buffer was measured using an auto well gamma counter (n = 4). The log *P* value was calculated by the logarithm of the ratio of radioactivity per milliliter (cpm/mL) in *n*-octanol to that in phosphate buffer and represents mean ± SD.

### 3.7. In Vitro Stability Experiments

The in vitro stability of [^125^I]**10** was examined in phosphate buffered saline (PBS) and mouse plasma. Briefly, [^125^I]**10** solution (50 µL) in a sealed tube containing 0.1 M PBS pH 7.4 (450 µL) was incubated at 37 °C for 1 and 24 h. After incubation, the purities of radiotracers were analyzed by RP-HPLC with mobile phase system water (A) and methanol (B), B: 70–100%, 20 min using a Cosmosil^®^ 5C_18_ MSII (4.6 ID × 150 mm) column, a flow rate 1 mL/min. The column temperature was kept at 40 °C.

The plasma was prepared by centrifugation of murine blood at 4 °C for 20 min at 100 rpm. The reaction was started by the addition of [^125^I]**10** (20 μL) in the plasma (180 µL). The mixture was incubated in a shaker at 300 rpm at 37 °C, which was carried out three times. Samples (100 µL) were taken at 0, 1 and 24 h and 100 µL acetonitrile, divided into five portions, was added for deproteinization of the plasma. The samples were left in the fridge for 15 min and then centrifuged at 4 °C for 15 min at 150 rpm. The supernatant was filtered through a pore size of 0.45 μm, then analyzed by RP-HPLC with a mobile phase gradient system water (A) and methanol (B), B: 70–100%, for 20 min using a Cosmosil^®^ 5C_18_ MSII (4.6 ID × 150 mm) column at a flow rate of 1 mL/min. The column temperature was maintained at 40 °C. Radioactivity was measured by an auto well gamma counter.

### 3.8. Cellular Uptake Studies

The NSCLC cell lines H1975 (1 × 10^5^ cells/well), H441 (1 × 10^5^ cells/well) and H3255 (2 × 10^5^ cells/well) were grown in medium supplemented with 10% FBS and penicillin (100 IU/mL) streptomycin (100 mg/mL) on 6-well culture plates at 37 °C in a humidified atmosphere with 5% CO_2_ for 24 h. Then, the cell culture was incubated in a serum-free medium containing [^125^I]**10** (3.7 kBq/well) for 4 h. The medium from each well was removed and the cells were washed with 1 mL of ice-cold PBS. Finally, all cell fractions were lysed twice with 0.5 mL of 1 M NaOH aqueous solution. The radioactivity of every fraction was determined by an auto well gamma counter. Total cell protein was determined using the Bicinchoninic Acid (BCA) Protein Assay Kit (Nacalai) protocol, which uses bovine serum albumin as a standard reference. In blocking experiments, inhibitors (CO-1686 and gefitinib with a final concentration of 100 µM) were combined with radiotracer and assessed using the same method mentioned above. All data were expressed as percent dose per milligram of protein (% dose/mg protein) ± SD.

### 3.9. Animals

Male ddY mice (27–30 g, 6 weeks old) and female BALB/c nu/nu mice (12–17 g, 4 weeks old) were purchased from Japan SLC Inc. (Hamamatsu, Japan). The mice were housed in a cage with free access to food and water maintained at a constant temperature (23–25 °C) with a 12 h light/dark cycle. To prepare tumor-bearing mice, female BALB/c nu/nu mice were implanted subcutaneously, with H1975 (5 × 10^6^ cells/100 μL) and H3255 (1 × 10^7^ cells/100 μL) in the left and right shoulder, respectively. The tumor reached palpable size 2- and 3-weeks post-inoculation. Animal experiments were conducted in accordance with laboratory guidelines for the care and use of animals of Kanazawa University. The animal handling protocol was approved by the Kanazawa University Animal Care Committee (approval number: AP-163766).

### 3.10. Biodistribution Studies

Mice were injected intravenously via the lateral tail vein with a saline solution of [^125^I]**10** (25 kBq, 100 μL) containing 1% tween-80 and 10% ethanol. The ddY mice were sacrificed at 10 min, 1, 4 and 24 h; meanwhile, the tumor-bearing mice were sacrificed at 1 and 4 h post injection. The selected tissues and organs were collected and weighed and their radioactivity was measured using an auto well gamma counter. In blocking experiments, female BALB/c *nu/nu* mice were intraperitoneally injected with CO-1686 (30 mg/kg) as a blocking agent 1 h prior to intravenous injection of [^125^I]**10** (25 kBq, 100 μL). The mice were sacrificed 1 h after the administration of radiotracer. The biodistribution data are expressed as percent injected dose per gram of tissue (% ID/g) along with the SD.

### 3.11. Statistical Analysis

All data were analyzed using GraphPad Prism 5.0. All values are presented as mean ± SD. Differences between groups were analyzed either by one-way ANOVA followed by a Tukey’s multiple-comparison test or Dunnett’s multiple-comparison test or two-tailed unpaired Student’s *t*-test. The level of statistical significance was set to a *p* < 0.05.

## 4. Conclusions

This study successfully synthesized a radiotracer labeled with ^125^I with high yield and purity. In vitro evaluation in NSCLC demonstrated that [^125^I]**10** exhibits high in vitro specificity to NSCLC cells with EGFR L858R/T790M mutations compared to that to wild-type EGFR. However, the in vivo study showed inadequate tumor uptake, resulting in a low tumor-to-blood ratio. Rapid hepatobiliary clearance due to high lipophilicity may have caused low intratumoral uptake. Therefore, in order to achieve high tumor uptake and low nonspecific binding, structural modifications need to be made that decrease lipophilicity without reducing affinity toward EGFR L858R/T790M.

## Figures and Tables

**Table 1:** The half maximal inhibitory concentration (IC_50_) after exposure of **10**, CO-1686 or gefitinib to non-small cell lung cancer (NSCLC) cell lines by 2-(2-methoxy-4-nitrophenyl)-3-(4-nitrophenyl)-5-(2,4-disulfophenyl)-2H tetrazolium monosodium salt (WST-8) assay.

Cell Lines	Mutation Status	IC_50_ (μM)		
		10	CO-1686	Gefitinib
H1975	L858R/T790M	0.20 ± 0.05	0.14 ± 0.05	>10
H3255	L858R	0.50 ± 0.21	0.15 ± 0.02	0.02 ± 0.02
H441	Wild-type	1.84 ± 0.44	0.26 ± 0.04	>10

Data represent the mean ± SD of three separate experiments.

**Table 2:** Inhibition activities to epidermal growth factor receptor (EGFR) tyrosine kinases (wild-type and L858R/T790M mutations).

Compound	IC_50_ Value (μM)		SI (WT: L858R/T790M)
	Wild-Type (WT)	L858R/T790M	
CO-1686	1.68 ± 0.15	0.04 ± 0.01	42
10	>10	0.31 ± 0.15	>32

Data represent the mean ± SD of three separate experiments.

**Table 3:** Biodistribution of [^125^I]**10** at 10 min, 1, 4 and 24 h after i.v. injection in ddY mice.

Tissues	Time after Injection			
	10 min	1 h	4 h	24 h
[^125^I]**10**	–	–	–	–
Blood	1.06 (0.19)	0.49 (0.05)	0.33 (0.07)	0.05 (0.01)
Liver	26.69 (0.81)	18.57 (2.72)	7.85 (0.92)	0.80 (0.10)
Kidney	7.48 (1.55)	3.76 (0.40)	2.37 (0.17)	0.25 (0.01)
Small intestine	9.46 (2.19)	23.96 (2.18)	9.81 (1.03)	0.10 (0.01)
Large intestine	0.57 (0.04)	0.53 (0.08)	53.44 (6.73)	0.34 (0.08)
Spleen	2.14 (0.50)	0.90 (0.17)	0.58 (0.11)	0.08 (0.04)
Pancreas	1.93 (0.37)	0.99 (0.26)	0.41 (0.03)	0.10 (0.11)
Lung	4.47 (1.39)	1.33 (0.08)	0.86 (0.10)	0.26 (0.03)
Heart	3.11 (0.64)	0.79 (0.13)	0.35 (0.09)	0.09 (0.05)
Stomach ^‡^	3.52 (2.70)	5.10 (1.18)	1.38 (0.56)	0.08 (0.02)
Bone	1.16 (0.21)	0.50 (0.22)	0.29 (0.11)	0.07 (0.05)
Muscle	1.40 (0.16)	0.44 (0.05)	0.13 (0.03)	0.04 (0.04)
Brain	0.11 (0.02)	0.05 (0.02)	0.03 (0.01)	0.01 (0.01)
Urine	–	–	–	4.07 (0.83)
Feces	–	–	–	69.93 (15.71)

Data were presented as % ID/g tissue. Each value represents mean ± SD for four mice. ^‡^ Presented as % ID/organ.

**Table 4:** Biodistribution of [^125^I]**10** at 1 and 4 h after i.v. injection in tumor-bearing mice.

Tissues	Time after Injection		
	1 h	4 h	Blocking (1 h)
[^125^I]**10**	–	–	–
Blood	1.74 (0.18)	0.77 (0.07)	1.79 (0.21)
Liver	28.12 (5.43)	12.40 (1.06)	21.77 (2.83)
Kidney	5.26 (1.50)	1.62 (0.26)	4.19 (1.28)
Small intestine	85.58 (7.68)	9.82 (3.17)	60.68 (6.67)
Large intestine	18.65 (19.88)	144.21 (10.97)	8.96 (7.48)
Spleen	3.20 (0.62)	0.71 (0.19)	2.81 (0.77)
Pancreas	1.99 (0.52)	0.49 (0.12)	3.87 (0.87)
Lung	4.67 (0.85)	1.02 (0.28)	3.40 (0.93)
Heart	1.46 (0.31)	0.60 (0.67)	1.53 (0.58)
Stomach ^‡^	1.04 (0.36)	0.48 (0.19)	1.44 (0.57)
Bone	0.50 (0.40)	0.24 (0.20)	1.07 (0.36)
Muscle	0.86 (0.21)	0.27 (0.08)	1.09 (0.42)
Brain	0.11 (0.08)	0.04 (0.02)	0.10 (0.02)
H1975	1.77 (0.43)	0.43 (0.08)	1.65 (0.64)
H3255	1.63 (0.23)	0.70 (0.13)	1.47 (0.71)

Data were presented as % ID/g tissue. Each value represents mean ± SD for four mice. ^‡^ Presented as % ID/organ.

## Supplementary Materials

The following are available online at https://www.mdpi.com/1420-3049/25/12/2914/s1, Figure S1: The proton and carbon NMR peak of 1-iodo-3,5-dinitrobenzene (**1**), Figure S2: The proton and carbon NMR peak of 5-iodobenzene-1,3-diamine (**2**), Figure S3: The proton and carbon NMR peak of tert-butyl (3-amino-5-iodophenyl)carbamate (**3**), Figure S4: The proton and carbon NMR peak of tert-butyl (3-((2-chloro-5-(trifluoromethyl)pyrimidin-4-yl)amino)-5-iodophenyl)carbamate (**4**), Figure S5: The proton and carbon NMR peak of *N*-(3-((2-chloro-5-(trifluoromethyl)pyrimidin-4-yl)amino)-5-iodophenyl) acrylamide (**5**), Figure S6: The proton and carbon NMR peak of 4-fluoro-2-methoxy-1-nitrobenzene (**6**), Figure S7: The proton and carbon NMR peak of 1-(piperazine-1-yl)ethan-1-one (**7**), Figure S8: The proton and carbon NMR peak of 1-(4-(3-methoxy-4-nitrophenyl)piperazine-1-yl)ethan-1-one (**8**), Figure S9: The proton NMR peak of 1-(4-(4-amino-3-methoxyphenyl)piperazine-1-yl)ethan-1-one (**9**), Figure S10: The proton and carbon NMR peak of *N*-(3-((2-((4-(4-acetylpiperazin-1-yl)-2-methoxyphenyl)amino)-5-(trifluoromethyl)pyrimidin-4-yl)amino)-5- iodophenyl)acrylamide (**10**), Figure S11: The proton NMR peak of *N*-(3-((2-((4-(4-acetylpiperazin-1-yl)-2- methoxyphenyl)amino)-5-(trifluoromethyl)pyrimidin-4-yl)amino)-5-(tributylstannyl) phenyl) acrylamide (**11**), Figure S12: The chromatograms of (a) nonradioactive iodinated compound **10** (ICO1686) and (b) radioactive compound [^125^I]**10** ([^125^I]ICO1686), Figure S13: The stability of radiolabeled compound [^125^I]**10** in PBS and plasma.
