# Supplementary material for: Synthesis and Fundamental Evaluation of Radioiodinated Rociletinib (CO-1686) as a Probe to Lung Cancer with L858R/T790M Mutations of Epidermal Growth Factor Receptor (EGFR)
Source: Molecules. 2020 Jun 24;25(12):2914. doi: 10.3390/molecules25122914 (PMC7356761; doi:10.3390/molecules25122914)
Supplement: Supplementary file 1 [file molecules-25-02914-s001.pdf]

Supporting Information

Synthesis and fundamental evaluation of radioiodinated rociletinib (CO-1686) as a probe to lung cancer with L858R/T790M mutations of epidermal growth factor receptor (EGFR)

Muammar Fawwaz[1,2], Kenji Mishiro[3], Ryuichi Nishii[4], Izumi Sawazaki[1], Kazuhiro Shiba[5], Seigo Kinuya[1] and Kazuma Ogawa[1,3]*

[1] Graduate School of Medical Sciences, Kanazawa University, Kakuma-machi, Kanazawa, Ishikawa 920-1192, Japan.
[2] Faculty of Pharmacy, Universitas Muslim Indonesia, Urip Sumoharjo KM. 10, Makassar 90-231, Indonesia.
[3] Institute for Frontier Science Initiative, Kanazawa University, Kakuma-machi, Kanazawa, Ishikawa 920-1192, Japan.
[4] National Institute of Radiological Sciences (NIRST), QST, Inage-ku, Chiba 263-8555, Japan.
[5] Advanced Science Research Center, Kanazawa University, Takara-machi, Kanazawa, Ishikawa 920-8640, Japan.

***Corresponding Author**

Institute for Frontier Science Initiative; Kanazawa University; Kakuma- machi, Kanazawa 920-1192; Japan.
Telephone: 81-76-234-4460; Fax: 81-76-234-4459
E-mail: kogawa@p.kanazawa-u.ac.jp

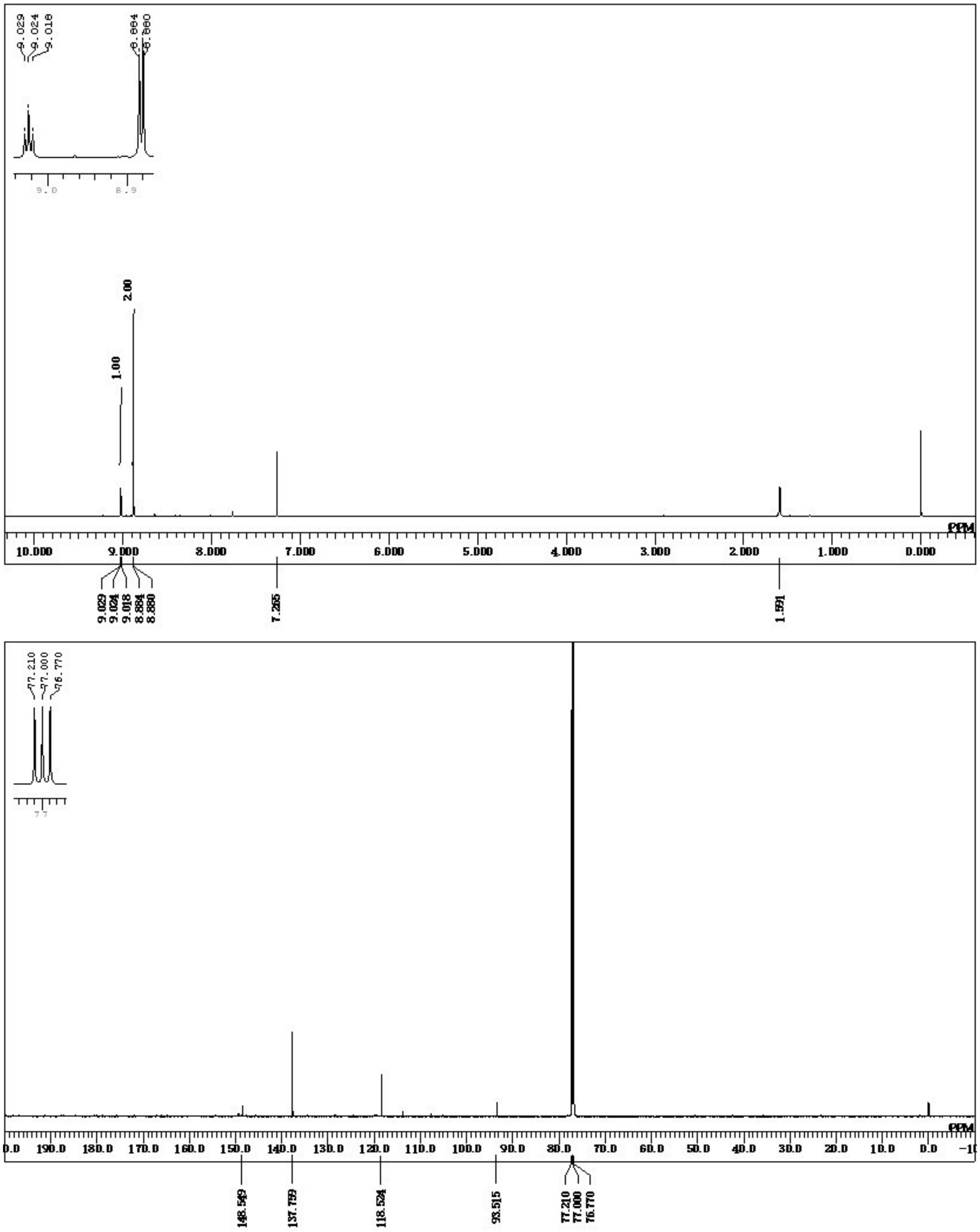

Figure S1. The proton and carbon NMR peak of 1-iodo-3,5-dinitrobenzene (**1**). ^{1}H NMR (400 MHz, $CDCl_3$): δ 8.88 (2H, d, *J* = 1.6 Hz), 9.02 (1H, t, *J* = 2.0 Hz). ^{13}C NMR (100 MHz, $CDCl_3$): δ 93.5, 118.5, 137.8, 148.5. HRMS (FAB+) calculated for $C_6H_3IN_2O_4$ $[M+ H]^+$: *m/z* = 294.9216, found 294.9225.

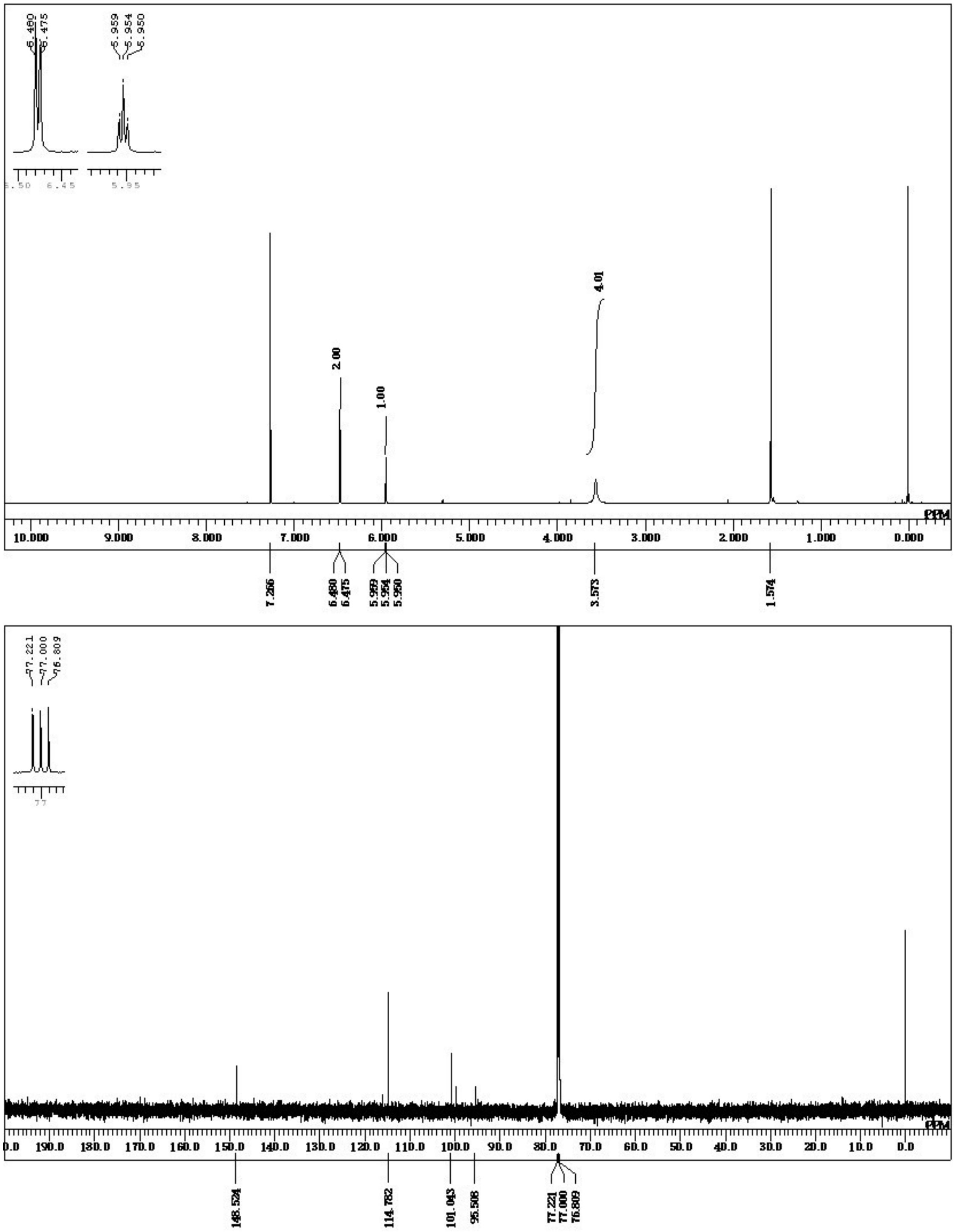

Figure S2. The proton and carbon NMR peak of 5-iodobenzene-1,3-diamine (**2**). ^{1}H NMR (400 MHz, $CDCl_3$): δ 3.57 (4H, s), 5.95 (1H, t, *J* = 2.0 Hz), 6.48 (2H, d, *J* = 2.0 Hz). ^{13}C NMR (100 MHz, $CDCl_3$): δ 95.5, 101.0, 114.8, 148.5. HRMS (FAB+) calculated for $C_6H_7IN_2$ $[M+ H]^+$: *m/z* = 233.9654, found 233.9645.

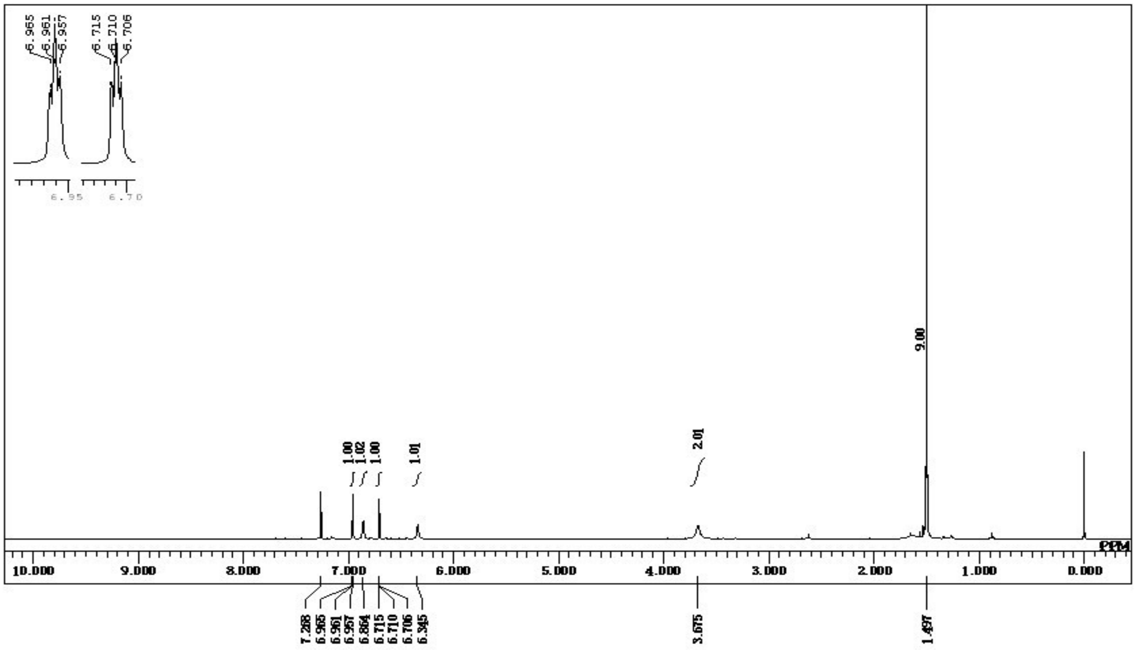

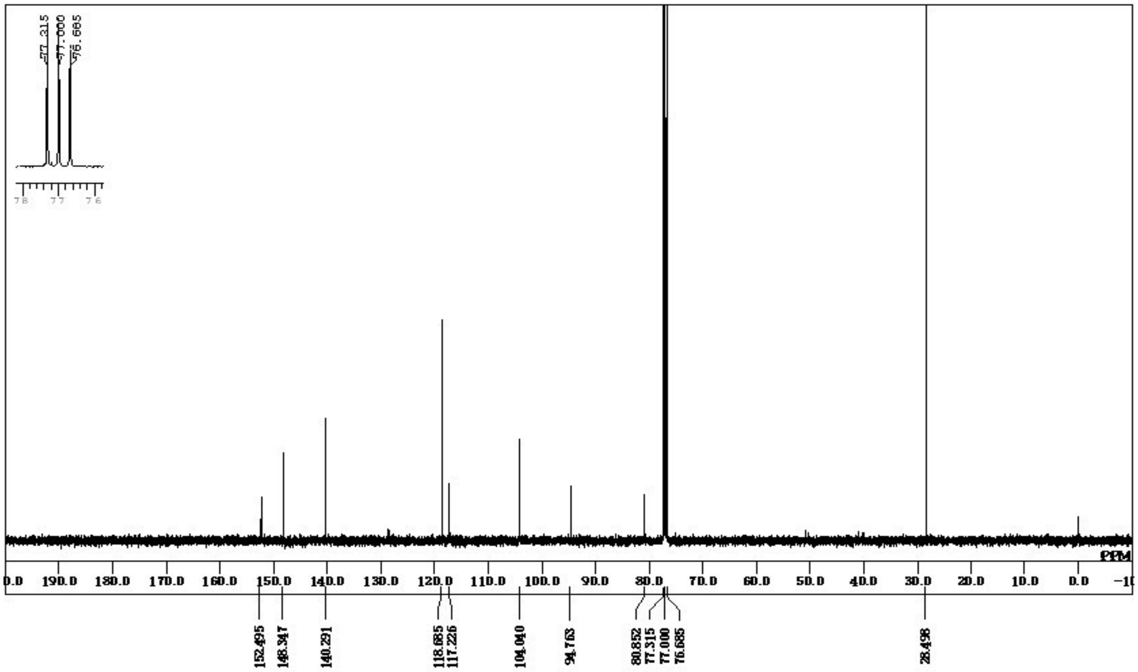

Figure S3. The proton and carbon NMR peak of tert-butyl (3-amino-5-iodophenyl)carbamate (**3**). ^{1}H NMR (400 MHz, $CDCl_3$): δ 1.50 (9H, s), 3.68 (2H, s), 6.34 (1H, s), 6.71 (1H, t, J = 1.6 Hz), 6.86 (1H, s), 6.96 (1H, t, J = 1.6 Hz). ^{13}C NMR (100 MHz, $CDCl_3$): δ 28.5 (3C), 80.9, 94.8, 104.0, 117.2, 118.7, 140.3, 148.3, 152.5. HRMS (FAB+) calculated for $C_{11}H_{15}IN_2O_2$ $[M+H]^+$: m/z = 334.0178, found 334.0168.

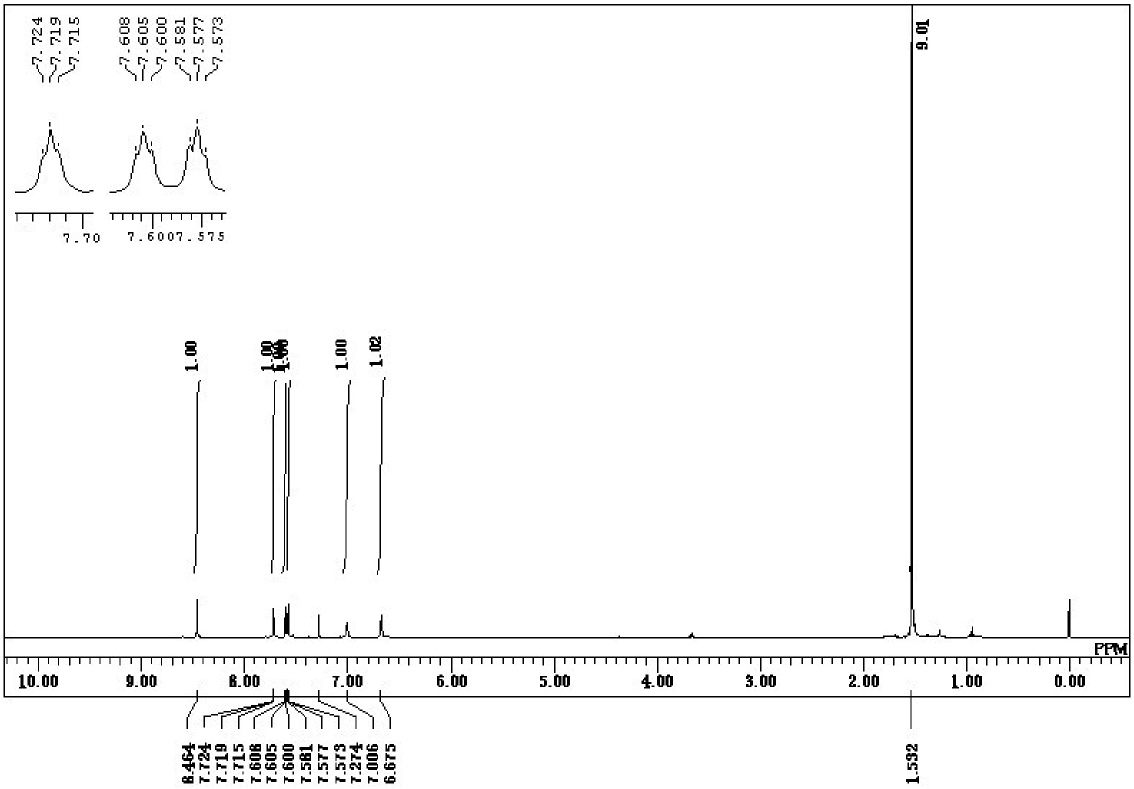

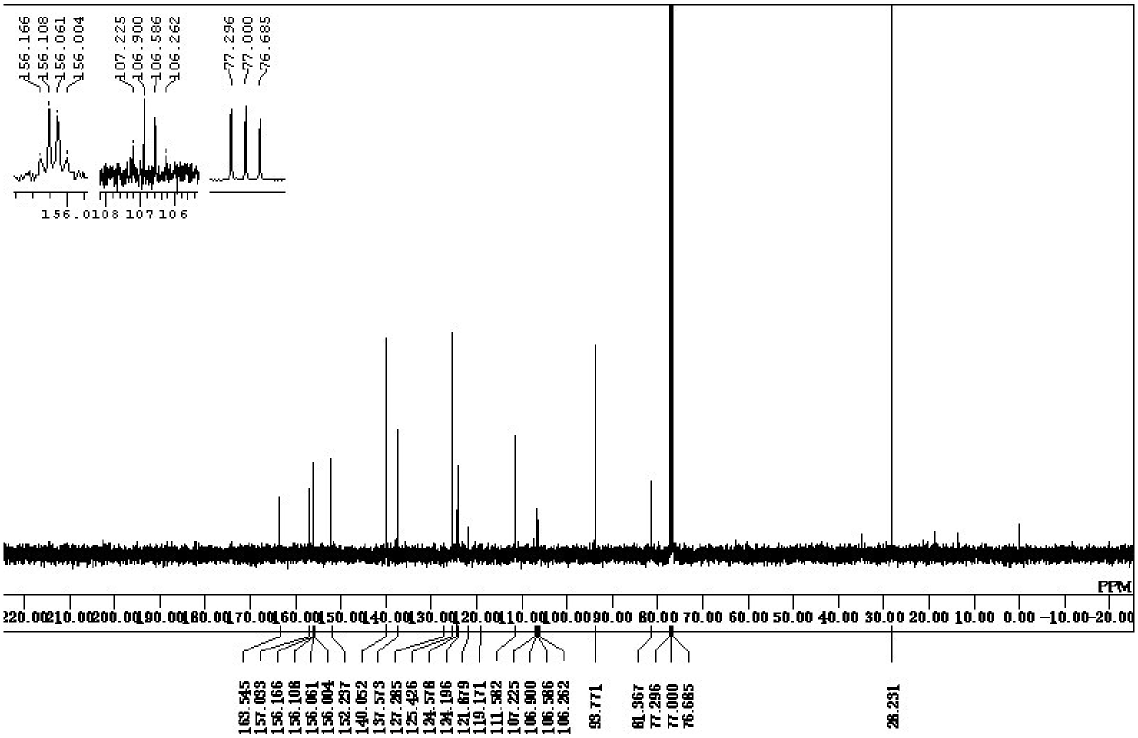

Figure S4. The proton and carbon NMR peak of tert-butyl (3-((2-chloro-5-(trifluoromethyl)pyrimidin-4-yl)amino)-5-iodophenyl)carbamate (**4**). ^{1}H NMR (400 MHz,

$CDCl_3$): δ 1.53 (9H, s), 6.68 (1H, s), 7.00 (1H, s), 7.58 (1H, t, J = 1.6 Hz), 7.61 (1H, t, J = 1.6 Hz), 7.72 (1H, t, J = 1.6 Hz), 8.46 (1H, s). ^{13}C NMR (100 MHz, $CDCl_3$): δ 28.2 (3C), 81.4, 93.8, 106.7 (q, J_{CF} = 31.4 Hz), 111.6, 123.0 (q, J_{CF} = 270 Hz), 124.2, 125.4, 137.6, 140.1, 152.2, 156.1 (q, J_{CF} = 4.7 Hz), 157.0, 163.5. HRMS (FAB+) calculated for $C_{16}H_{15}ClF_3IN_4O_2$ $[M+ H]^+$: m/z = 513.9880, found 513.9875.

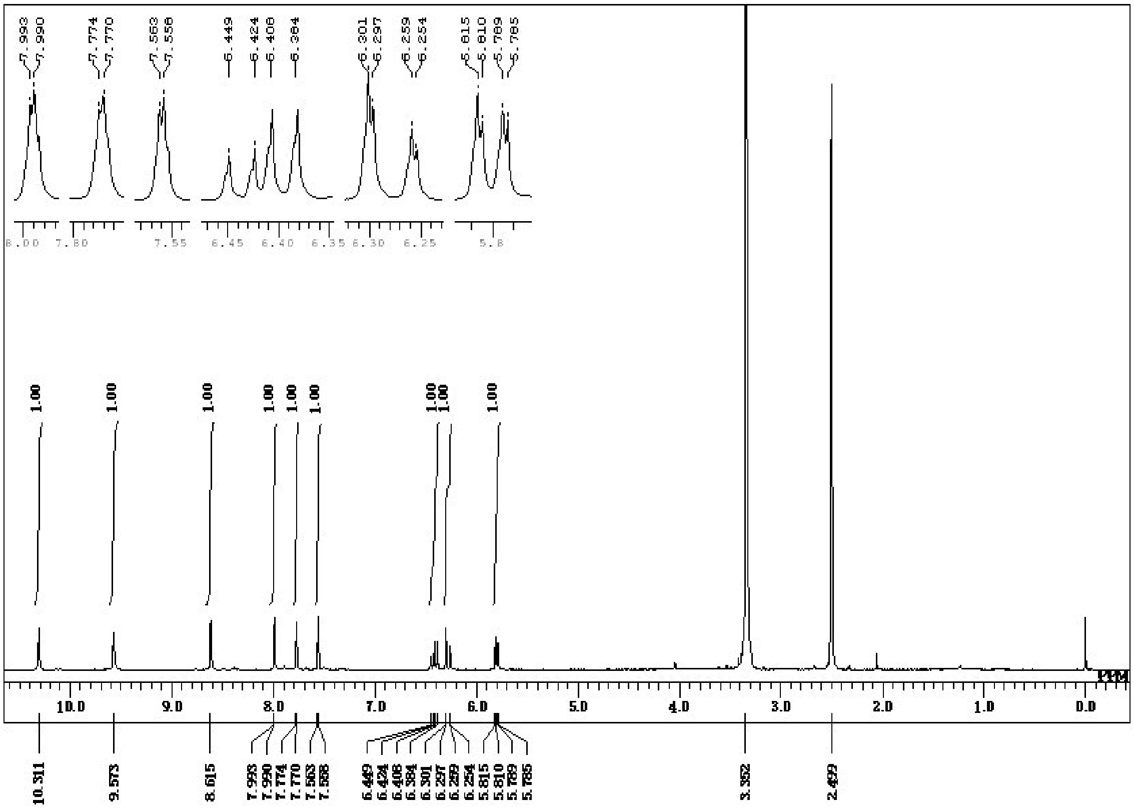

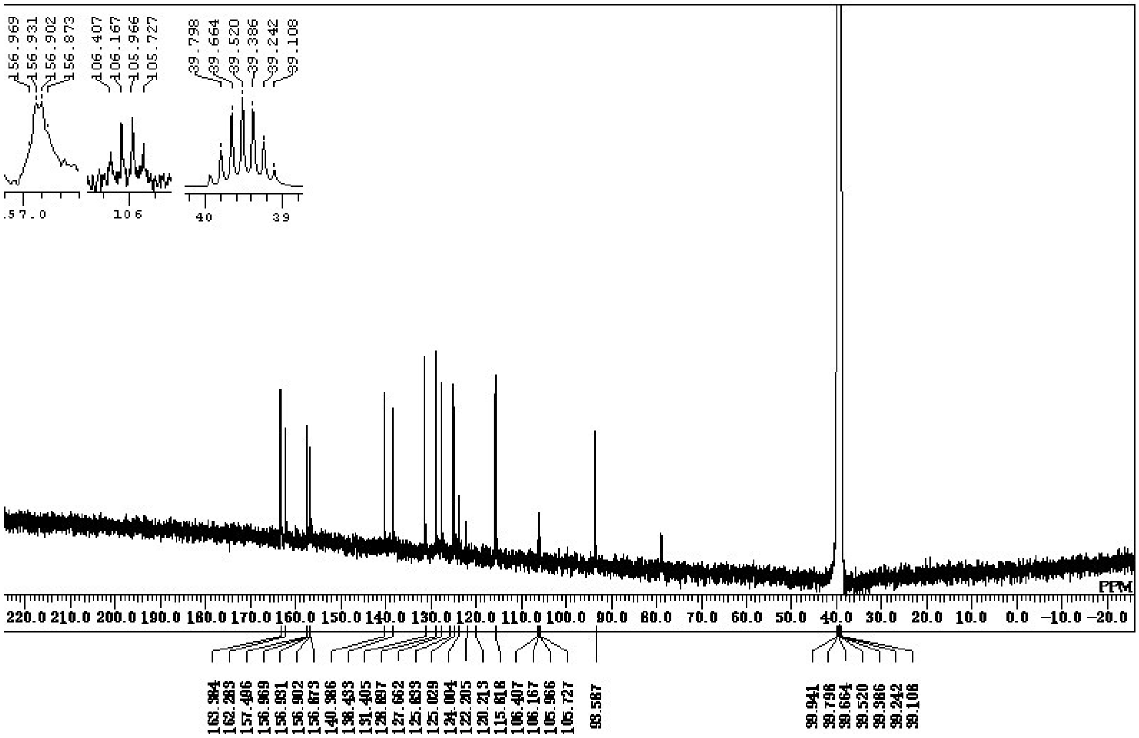

Figure S5. The proton and carbon NMR peak of N-(3-((2-chloro-5-(trifluoromethyl)pyrimidin-4-yl)amino)-5-iodophenyl) acrylamide (**5**). ^{1}H NMR (400

MHz, $(CD_3)_2SO$): δ 5.79 (1H, dd, J = 10.0, 2.0 Hz), 6.27 (1H, dd, J = 16.8, 2.0 Hz), 6.40 (1H, dd, J = 16.4, 10.0 Hz), 7.56 (1H, d, J = 2.0 Hz), 7.77 (1H, d, J = 2.0 Hz), 7.99 (1H, d, J = 2.0 Hz), 8.62 (1H, s), 9.57 (1H, s), 10.31 (1H, s). ^{13}C NMR (150 MHz, $(CD_3)_2SO$): δ 93.6, 106.1 (q, J_{CF} = 30.2 Hz), 115.8, 123.0 (q, J_{CF} = 270 Hz), 125.0, 127.7, 128.9, 131.4, 138.4, 140.4, 156.9 (q, J_{CF} = 4.4 Hz), 157.5, 162.3, 163.4. HRMS (FAB+) calculated for $C_{14}H_9ClF_3IN_4O$ $[M+H]^+$: m/z = 468.9461, found 468.9559.

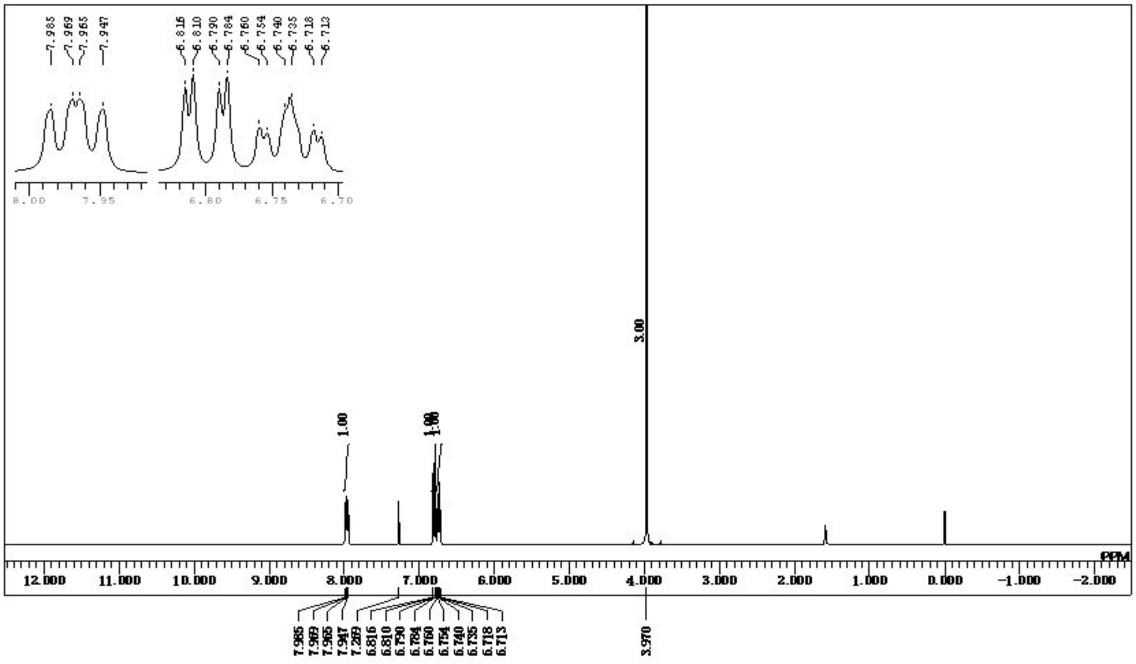

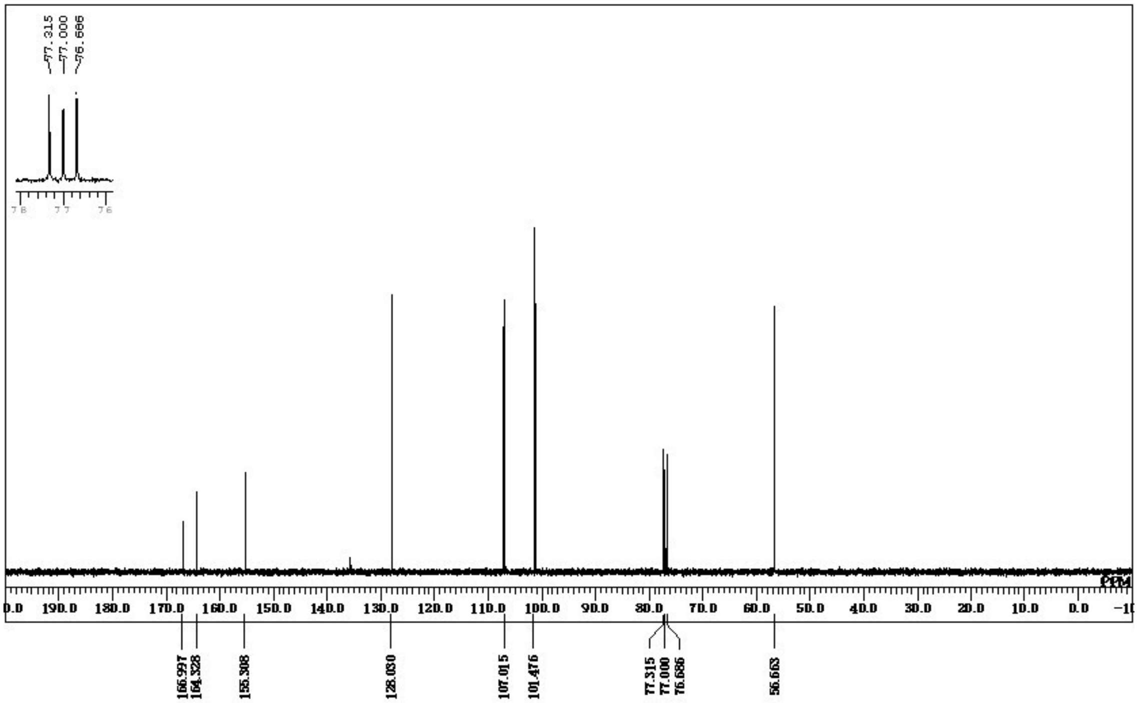

Figure S6. The proton and carbon NMR peak of 4-fluoro-2-methoxy-1-nitrobenzene (**6**). ^{1}H NMR (400 MHz, $CDCl_3$): δ 3.97 (3H, s), 6.74 (1H, dt, J = 8.4, 2.0 Hz), 6.80 (1H, dd, J = 10.4, 2.4 Hz), 7.96 (1H, dd, J = 8.4, 6.4 Hz). ^{13}C NMR (100 MHz, $(CD_3)_2SO$): δ 56.7, 101.5, 107.0, 128.0, 155.3, 164.3, 167.0. HRMS (DART+) calculated for $C_7H_6FNO_3$ $[M+H]^+$: m/z = 172.0331, found 172.0345.

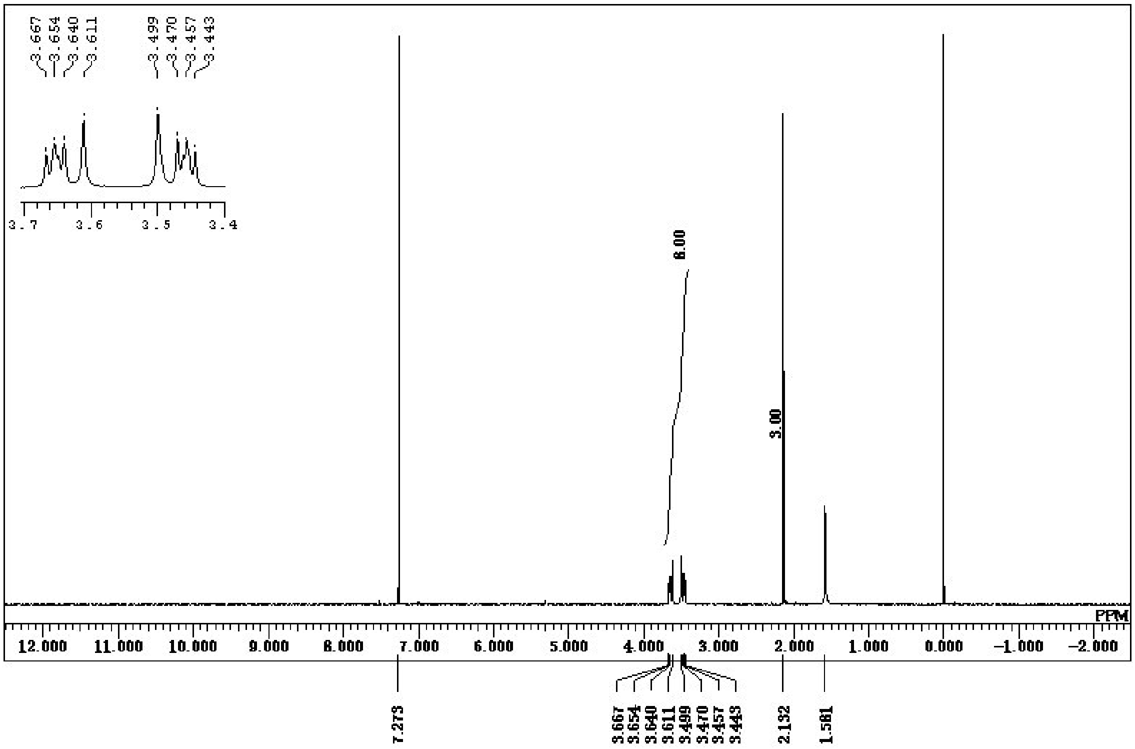

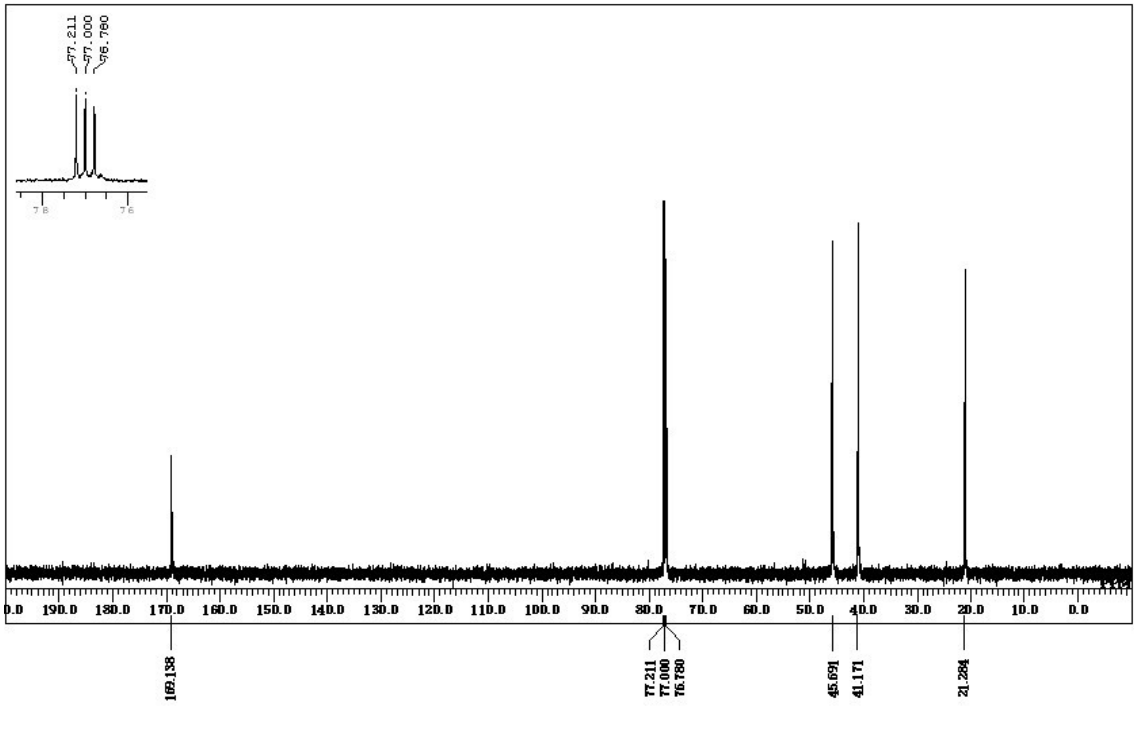

Figure S7. The proton and carbon NMR peak of 1-(piperazine-1-yl)ethan-1-one (**7**). ^{1}H NMR (400 MHz, $CDCl_3$): δ 2.13 (3H, s), 3.40-3.70 (8H, m). ^{13}C NMR (100 MHz, $CDCl_3$): δ 21.3, 41.2 (2C), 45.7 (2C), 169.1. HRMS (FAB+) calculated for $C_6H_{12}N_2O$ $[M+ H]^{+}$: *m/z* = 129.0949, found 129.1031.

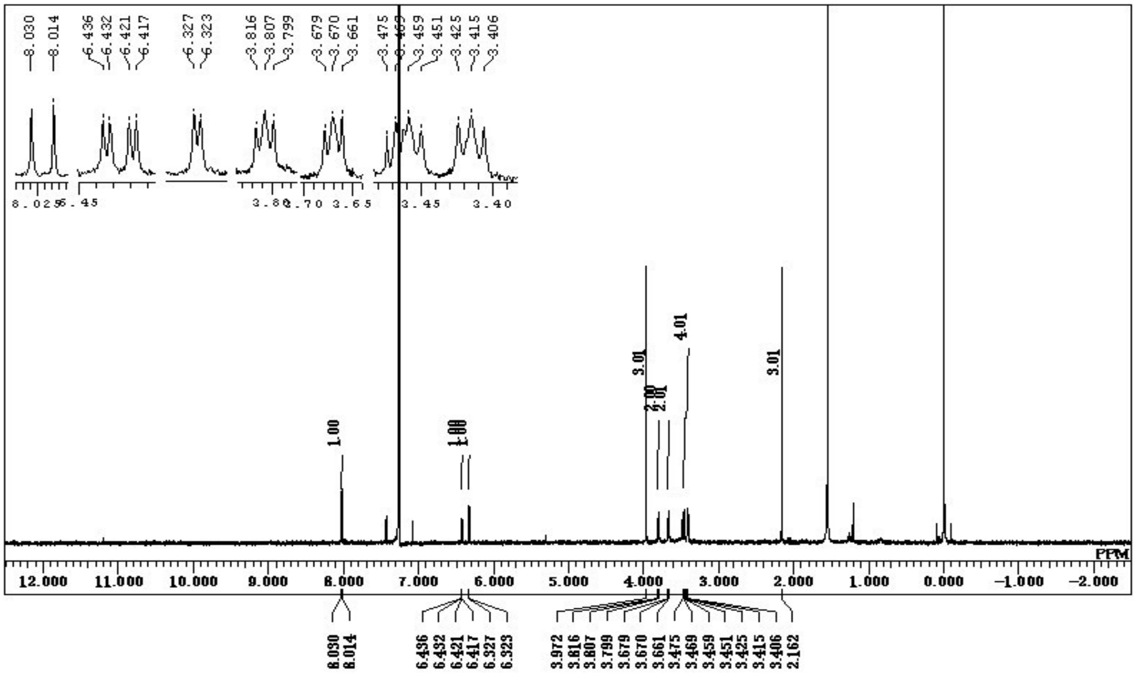

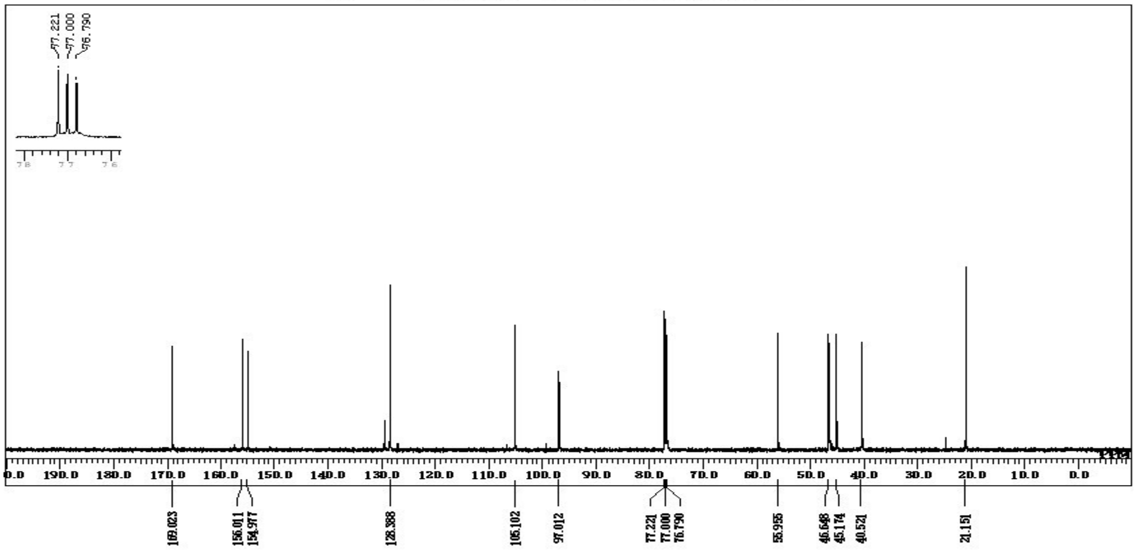

Figure S8. The proton and carbon NMR peak of 1-(4-(3-methoxy-4-nitrophenyl)piperazine-1-yl)ethan-1-one (**8**). ^{1}H NMR (400 MHz, $CDCl_3$): δ 2.16 (3H, s), 3.40-3.50 (4H, m), 3.67 (2H, t, *J* = 4.0 Hz), 3.81 (2H, t, *J* = 4.0 Hz), 3.97 (3H, s), 6.33 (1H, d, *J* = 1.6 Hz), 6.42 (1H, dd, *J* = 6.0, 1.6 Hz), 8.02 (1H, d, *J* = 6.4 Hz). ^{13}C NMR (100 MHz, $CDCl_3$): δ, 21.2, 40.5, 45.2, 46.5, 46.6, 56.0, 97.0, 105.1, 128.4, 129.5, 155.0, 156.0, 169.0. HRMS (FAB+) calculated for $C_{13}H_{17}N_3O_4$ $[M+H]^+$: *m/z* = 280.1219, found 280.1295.

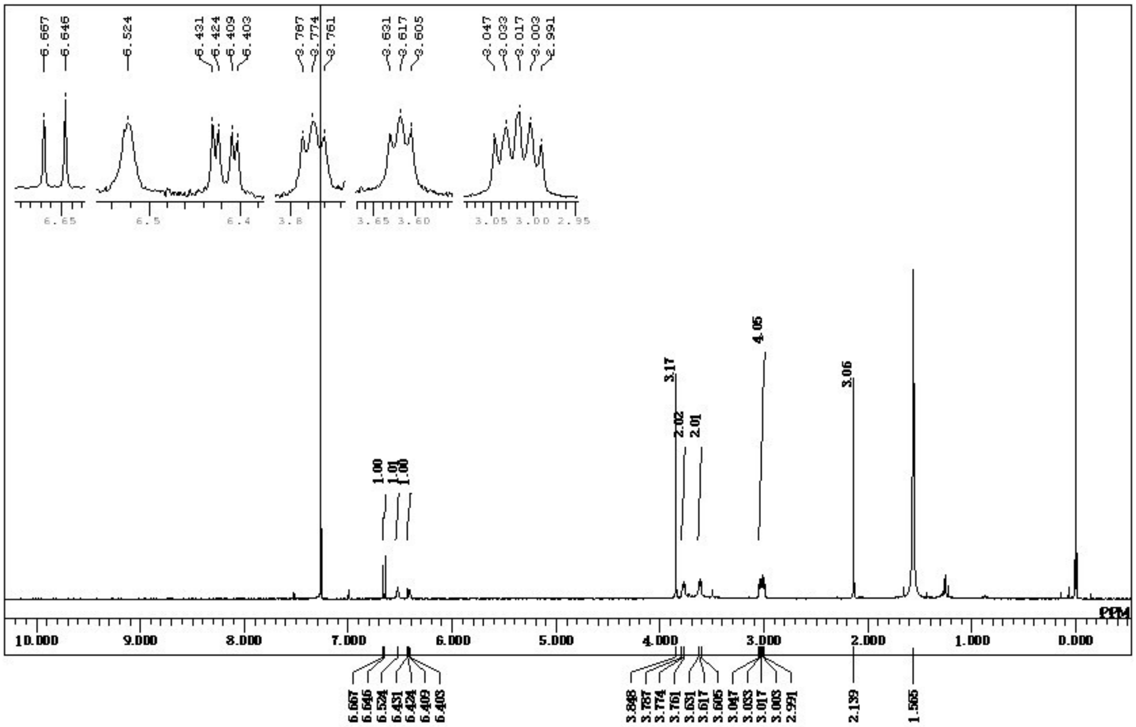

Figure S9. The proton NMR peak of 1-(4-(4-amino-3-methoxyphenyl)piperazine-1-yl)ethan-1-one (**9**). ^{1}H NMR (400 MHz, $CDCl_3$): δ 2.13 (3H, s), 2.95-3.08 (4H, m), 3.62 (2H, t, J = 5.6 Hz), 3.77 (2H, t, J = 5.2 Hz), 3.85 (3H, s), 6.42 (1H, dd, J = 8.8, 2.8 Hz), 6.52 (1H, s), 6.66 (1H, d, J = 8.4 Hz). HRMS (FAB+) calculated for $C_{13}H_{19}N_3O_2$ [M+H]$^+$: m/z = 249.1477, found 249.1464.

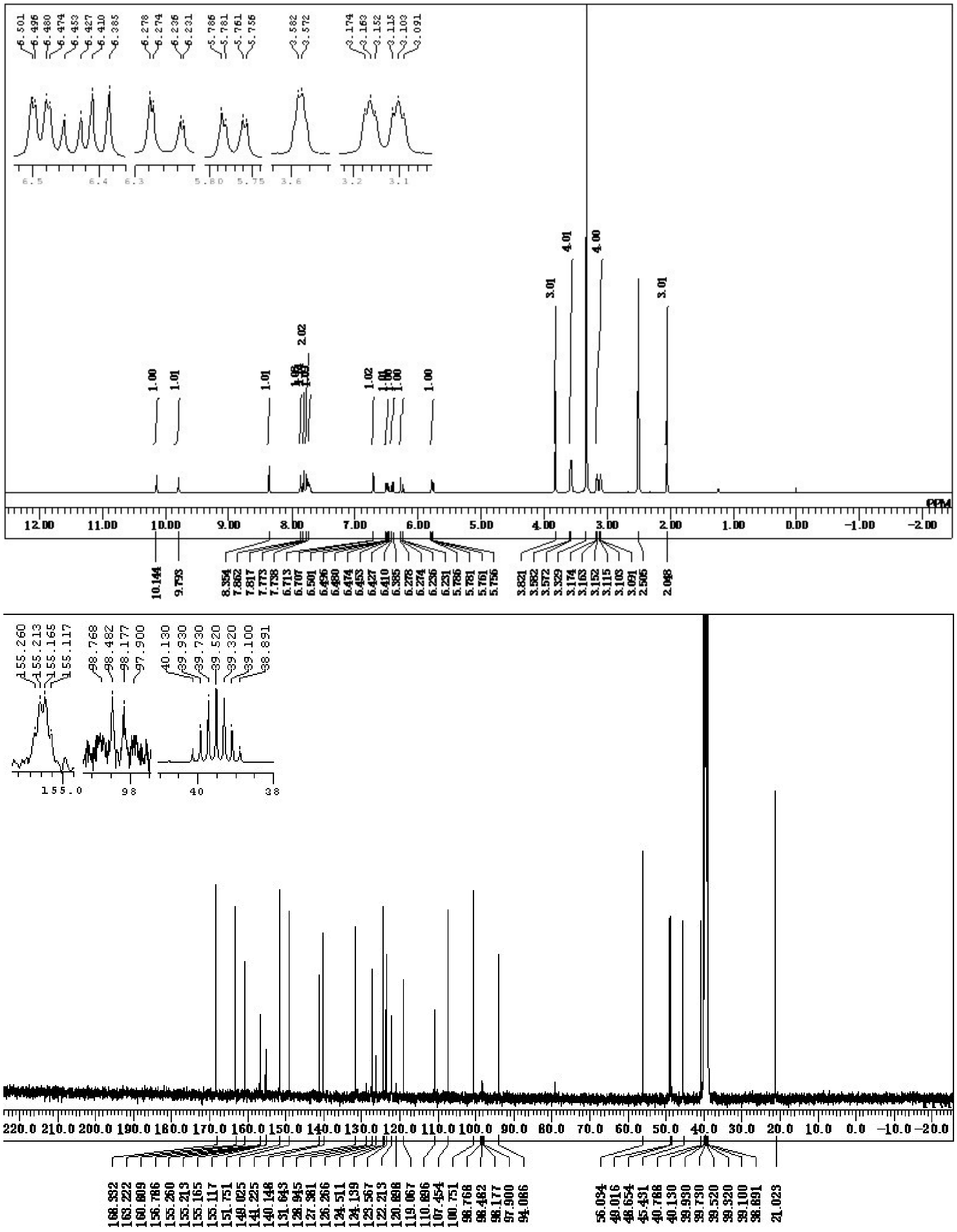

Figure S10. The proton and carbon NMR peak of N-(3-((2-((4-(4-acetylpiperazin-1-yl)-2-methoxyphenyl)amino)-5-(trifluoromethyl)pyrimidin-4-yl)amino)-5-iodophenyl)acrylamide (**10**). ^{1}H NMR (400 MHz, $(CD_3)_2SO$): δ 2.05 (3H, s), 3.10 (2H, t, *J* = 4.8 Hz), 3.16 (2H, t, *J* = 4.4 Hz), 3.53-3.63 (4H, m), 3.82 (3H, s), 5.77 (1H, dd, *J*

= 10.0, 2.0 Hz), 6.25 (1H, dd, *J* = 16.8, 2.0 Hz), 6.41 (1H, dd, *J* = 16.8, 10.0 Hz), 6.49 (1H, dd, *J* = 8.8, 2.0 Hz), 6.71 (1H, s), 7.74 (1H, s), 7.77 (2H, s), 7.82 (1H, s), 7.86 (1H, s), 8.35 (1H, s), 9.79 (1H, s), 10.14 (1H, s). ^{13}C NMR (100 MHz, $(CD_3)_2SO$): δ 21.0, 40.8, 45.4, 48.7, 49.0, 56.0, 94.1, 98.3 (q, J_{CF} = 30.5 Hz), 100.8, 107.5, 110.9, 119.1, 122.2, 124.1, 124.5, 124.8 (q, J_{CF} = 270 Hz), 127.4, 131.6, 140.1, 141.2, 149.0, 151.8, 155.2 (q, J_{CF} = 4.8 Hz), 156.8, 160.8, 163.2, 168.3. HRMS (FAB+) calculated for $C_{27}H_{27}F_3IN_7O_3$ $[M+ H]^+$: *m/z* = 681.1172, found 681.1186.

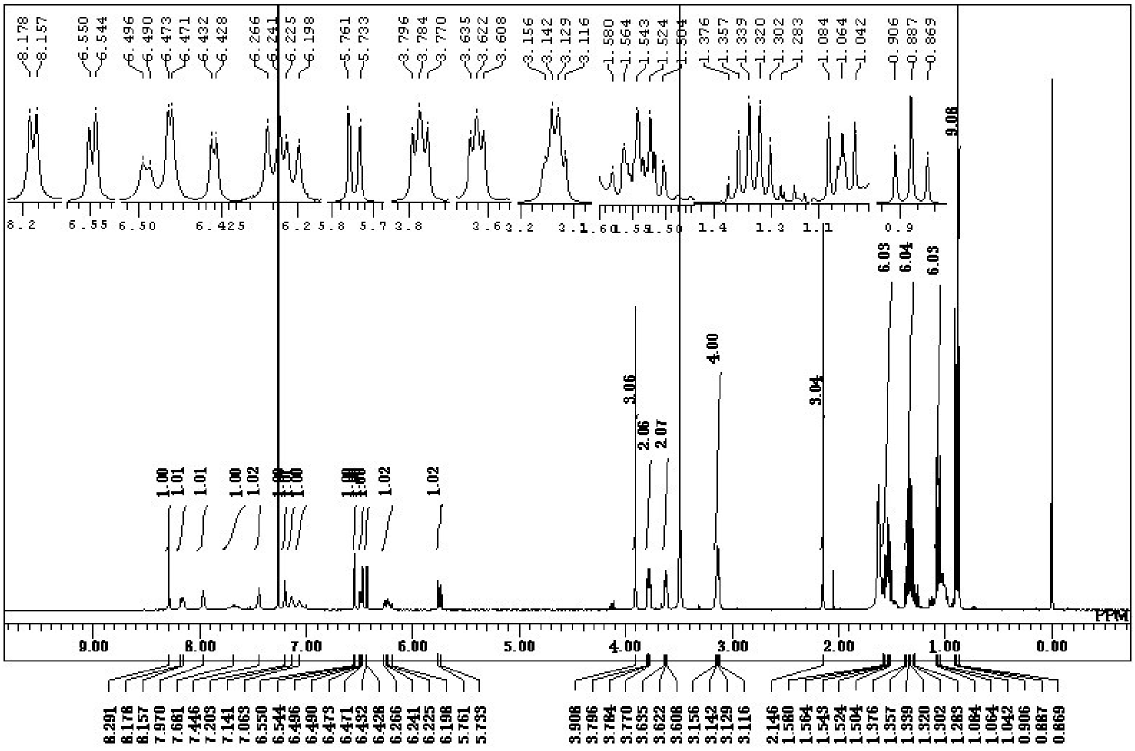

Figure S11. The proton NMR peak of N-(3-((2-((4-(4-acetylpiperazin-1-yl)-2-methoxyphenyl)amino)-5-(trifluoromethyl)pyrimidin-4-yl)amino)-5-(tributylstannyl) phenyl) acrylamide (**11**). ^{1}H NMR (400 MHz, ($CDCl_3$): δ 0.88 (9H, t, *J* = 7.6 Hz), 1.06 (6H, t, *J* = 8.0 Hz), 1.33 (6H, sex, *J* = 7.6 Hz), 1.54 (6H, quin, *J* = 7.6 Hz), 2.14 (3H, s), 3.10-3.20 (4H, m), 3.62 (2H, t, *J* = 5.6 Hz), 3.78 (2H, t, *J* = 5.6 Hz), 3.91 (3H, s), 5.75 (1H, d, *J* = 11.2 Hz), 6.23 (1H, dd, *J* = 17.2, 10.8 Hz), 6.43 (1H, d, *J* = 1.6 Hz), 6.48 (1H, dd, *J* = 7.6, 2.0 Hz), 6.54 (1H, d, *J* = 2.4 Hz), 7.68 (1H, s) 7.06 (1H, s), 7.14 (1H, s), 7.20 (1H, s), 7.44 (1H, s), 7.97 (1H, s), 8.16 (1H, d, *J* = 8.4 Hz), 8.29 (1H, s). HRMS (FAB+) calculated for $C_{39}H_{54}F_3N_7O_3Sn$ $[M+H]^+$: *m/z* = 845.3267 found 845.3262.

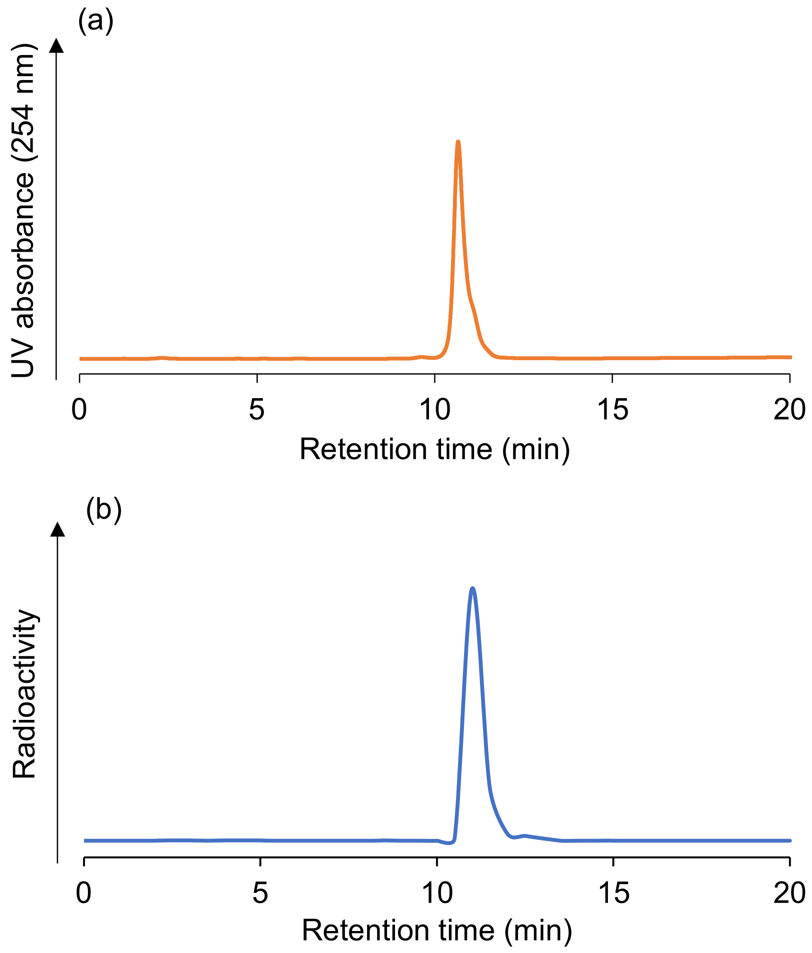

Figure S12. The chromatograms of (a) nonradioactive iodinated compound **10** (ICO1686) and (b) radioactive compound [^{125}I]**10** ([^{125}I]ICO1686).

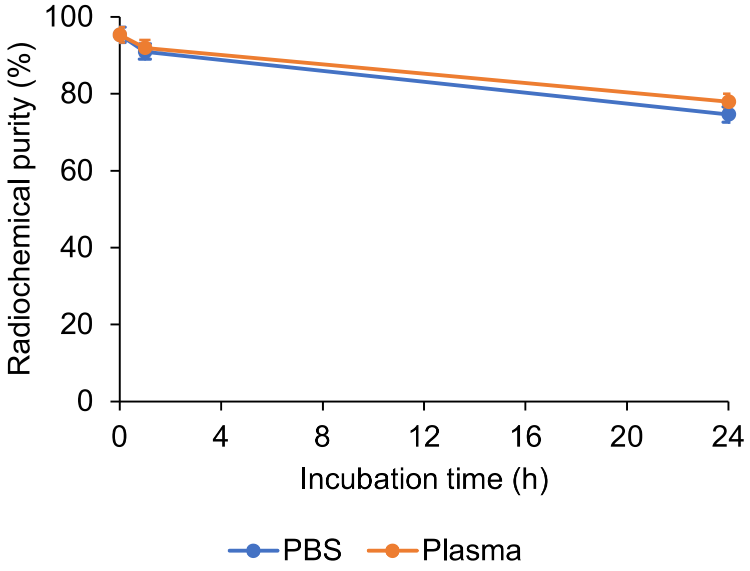

Figure S13. The stability of radiolabeled compound [^{125}I]**10** in PBS and plasma.
